# Mitochondria to plasma membrane redox signaling is essential for fatty acid β-oxidation-driven insulin secretion

**DOI:** 10.1016/j.redox.2024.103283

**Published:** 2024-07-23

**Authors:** Martin Jabůrek, Eduardo Klöppel, Pavla Průchová, Oleksandra Mozheitova, Jan Tauber, Hana Engstová, Petr Ježek

**Affiliations:** Department of Mitochondrial Physiology, No.75, Institute of Physiology of the Czech Academy of Sciences, Vídeňská 1083, Prague, 14220, Czech Republic

**Keywords:** *Redox signaling*, *Pancreatic β-cells*, *Fatty acid-stimulated insulin secretion*, *Redox-activated phospholipase iPLA2γ*, *Mitochondrial fatty acids*, *GPR40*

## Abstract

We asked whether acute redox signaling from mitochondria exists concomitantly to fatty acid- (FA-) stimulated insulin secretion (FASIS) at low glucose by pancreatic β-cells. We show that FA β-oxidation produces superoxide/H_2_O_2_, providing: *i*) mitochondria-to-plasma-membrane redox signaling, closing K_ATP_-channels synergically with elevated ATP (substituting NADPH-oxidase-4-mediated H_2_O_2_-signaling upon glucose-stimulated insulin secretion); *ii*) activation of redox-sensitive phospholipase iPLA_2_γ/PNPLA8, cleaving mitochondrial FAs, enabling metabotropic GPR40 receptors to amplify insulin secretion (IS). At fasting glucose, palmitic acid stimulated IS in wt mice; palmitic, stearic, lauric, oleic, linoleic, and hexanoic acids also in perifused pancreatic islets (PIs), with suppressed 1st phases in iPLA_2_γ/PNPLA8-knockout mice/PIs. Extracellular/cytosolic H_2_O_2_-monitoring indicated knockout-independent redox signals, blocked by mitochondrial antioxidant SkQ1, etomoxir, CPT1 silencing, and catalase overexpression, all inhibiting FASIS, keeping ATP-sensitive K^+^-channels open, and diminishing cytosolic [Ca^2+^]-oscillations. FASIS in mice was a postprandially delayed physiological event. Redox signals of FA β-oxidation are thus documented, reaching the plasma membrane, essentially co-stimulating IS.

## Highlights

• Fatty acids stimulate insulin secretion at low glucose in pancreatic islet β-cells.
• Mitochondrial β-oxidation of fatty acids provides redox signal for insulin secretion.
• Pancreatic islets release H_2_O_2_ upon insulin fatty acid-stimulated insulin secretion.
• Redox-activated mitochondrial phospholipase iPLA2γ supplies fatty acids for GPR40.
• Insulin is secreted up to 6 h after administration of Intralipid to mice.

## Graphical abstract

**Figure:**
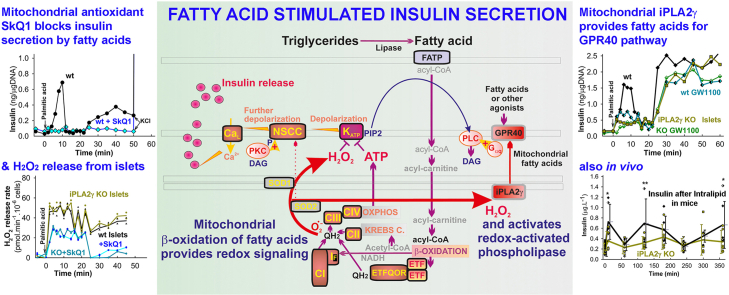

## Abbreviations

AUC –: area under curve
BCKAs –: branched-chain ketoacids
BSA –: bovine serum albumin
r-BEL –: r-bromoenol-lactone
CPT1 –: carnitine palmitoyltransferase 1
CRAT –: carnitine O-acetyltransferase
DAG –: diacylglycerols
DCF –: 2′,7′-dichlorodihydrofluorescein diacetate
Decyl-TPP –: decyltriphenylphosphonium
ETFQOR – electron-transfer flavoprotein: ubiquinone-oxido-reductase
FA –: fatty acid
FASIS –: fatty acid - stimulated insulin secretion
GPR40 –: G-protein-coupled receptor-40
GSIS –: glucose - stimulated insulin secretion
HRP –: horseradish peroxidase
IGVs –: insulin granule vesicles
iPLA_2_γ/PNPLA8 –: mitochondrial redox-sensitive phospholipase A_2_, isoform γ/patatin-like phospholipase domain-containing protein
K_ATP_ –: ATP-sensitive K^+^-channel
MAGs –: monoacylglycerols
NOX4 –: NADPH oxidase, isoform 4
NSCC –: nonspecific calcium channels
OXPHOS –: oxidative phosphorylation
PA –: palmitic acid
PIP_2_ –: phosphatidylinositol 4,5-bisphosphate
PNPLA8/iPLA_2_γ –: patatin-like phospholipase domain-containing protein/mitochondrial redox-sensitive phospholipase A_2_, isoform γ
ROI –: regions of the interest
ROS –: reactive oxygen species
s-BEL –: s-bromoenol-lactone
TALENs –: transcription activator-like effector nucleases
TGs –: triglycerides
TRPM –: transient receptor potential melastatin (channel)
UCP2 –: uncoupling protein 2

## 1 Introduction

Redox signaling from mitochondria [[1], [2], [3]] has been observed and documented in cases such as transcriptome reprogramming upon hypoxic cell adaptation [[4], [5], [6]], triggering redox-sensitive gene-regulatory processes, enabling progression through the S-phase of the cell cycle [7], and even contributing to local synaptic activity [8]. Mitochondrial reactive oxygen species (ROS) regulate the quiescence, activation, proliferation, and differentiation of stem cells [9], and were implicated in pathological reperfusion heart injury [10] when released excessively. Also, physiological ROS levels drive brown adipose tissue thermogenesis [11] and NLRP3-inflammasome assembly in macrophages [12]. ROS of mitochondrial origin, such as resulting from the addition of monooleoyl-glycerol [13], have been suggested to modulate insulin secretion [14]. An effect of antioxidants upon exhausted glutathione has been reported [15] as an unspecified link between glucose-stimulated insulin secretion (GSIS) and external H_2_O_2_.

Indeed, free fatty acids (FAs) are regarded as somewhat two-faced towards pancreatic β-cells, either being thought to augment GSIS [[16], [17], [18], [19]]; or in contrast, to suppress insulin secretion, when saturated FAs are in chronic excess and activate the toll-like receptors TLR2 and TLR4 [17,18,[20], [21], [22]]. Long-chain FAs (C12–C22), both saturated and unsaturated [[23], [24], [25], [26]], physiologically activate the G-protein-coupled receptor-40 (GPR40), *i.e*. the metabotropic receptor pathway [[23], [24], [25], [26], [27], [28], [29], [30], [31], [32], [33], [34]], reportedly potentiating GSIS [[16], [17], [18], [19]].

The existence of FA-stimulated insulin secretion (FASIS) [23] has not been widely recognized at low glucose concentrations (but cf. Ref. [20,[34], [35], [36], [37], [38], [39], [40]]), which otherwise does not stimulate insulin release alone. The strict dependence on intermediate/high glucose could match the dependence of the GPR40-pathway [[23], [24], [25], [26], [27], [28], [29], [30], [31], [32], [33], [34]] on the preceding high-frequency cytosolic [Ca^2+^]_c_-oscillations [[39], [40], [41], [42]], provided by voltage-dependent Ca^2+^-channels (Ca_V_; mostly Ca_L_) and voltage-gated K^+^ channels (K_V_), which are responsible for ascending and descending action potential, respectively. Hypothetically, FA β-oxidation at low glucose should produce sufficient ATP and superoxide/H_2_O_2_ to close ATP-sensitive K^+^ channels (K_ATP_) [36,[39], [40], [41], [42]]. The GPR40-FASIS branch would simultaneously amplify the basic metabolic K_ATP_-triggered FASIS component.

Upon GSIS, the transient receptor potential melastatin-2 (TRPM2) channel or other nonspecific calcium (NSCC) or Cl^**−**^ channels synergize with the 100 % closure of the K_ATP_ population [[43], [44], [45]]. This synergy is essentially required for a sufficient plasma membrane depolarization to **–**50 mV, which opens Ca_V_ channels [22,[39], [40], [41], [42], [43], [44], [45]]. Intermittently, K_V_ terminate the Ca_V_ conductance and resulting pulsatile Ca^2+^ influx into the β-cell cytosol causes [Ca^2+^]_c_ oscillations, transferred to pulsatile exocytosis of insulin granule vesicles (IGVs) [41]. [Ca^2+^]_c_ oscillations exhibit lag phases at intermediate glucose but are sustained at high glucose. The amount of time-integrated [Ca^2+^]_c_ determines the number of IGVs, hence the amount of secreted insulin [[39], [40], [41], [42], [43], [44], [45]].

The inability of GPR40 to stimulate insulin secretion by interacting with free FAs at low glucose would suggest that the β-oxidation component is insufficient to close 100 % of the K_ATP_ population and/or activate NSCCs [22,36,[39], [40], [41]]. However, a profound „experimental“ FASIS was demonstrated at 3 mM [37] and 5.5 mM [34,36] glucose, respectively. Since GPR40 silencing or the GPR antagonist GW1100 blocked ∼66 % of the accumulated insulin release in INS-1E cells responding to 15 μM palmitic acid at 3 mM glucose [37], the metabolic (non-amplified) FASIS component should account for the remaining ∼34 %.

How does this experimental set-up reflect *in vivo* conditions? Besides the intracellular hydrolysis of triglycerides (TGs) to diacyl- and monoacyl-glycerols (DAG, MAG), free long-chain FAs appear within PI capillaries in TGs contained in incoming chylomicrons, after cleavage by lipoprotein lipase [35]. Experimentally, an instant glucose administration (GSIS) evokes a nearly instantaneous 1st phase (5–10 min) and long-lasting (>1 h) 2nd phase of blood insulin elevation and glycemia elevation, returning to the fasting insulin and glycemia levels (5.5–6 mM) after 60–90 min in mice (e.g. Ref. [36]). However, typical postprandial responses to meal glycolytic metabolites are slightly more delayed, questioning the existence of a physiological 1st phase [41]. Moreover, postprandial chylomicrons, rich in TGs, come to the pancreas frequently, at least after 1 h in rodents and a few hours in humans [46], hence already under conditions of low blood glucose, approaching fasting glycemia. These facts are recalled for FASIS studies at low or intermediate glucose [23].

Using rat pancreatic β-cells INS-1E, we demonstrated that externally delivered palmitic acid was insufficient to activate GPR40 and did not stimulate FASIS upon siRNA-ablation of the mitochondrial redox-sensitive phospholipase A_2_ (iPLA_2_γ/PNPLA8) [37]. Elevated superoxide/H_2_O_2_ formation due to FA β-oxidation was required to activate this redox-sensitive iPLA_2_γ, which subsequently cleaves FAs from mitochondrial phospholipids and perpetuates FA β-oxidation and additional superoxide/H_2_O_2_ production. Resulting diffusing free FAs also stimulated GPR40-receptors. We have even monitored such FA diffusion up to the plasma membrane [37]. Hence the signal of lipid intake is amplified, when directed to GPR40. The remaining ∼30 % insulin release in iPLA_2_γ-deficient INS-1E cells probably proceeds due to FA β-oxidation *via* the canonical K_ATP_-Ca_V_-pathway of insulin secretion [36,37,[39], [40], [41], [42],^47^], even though UCP2-iPLA_2_γ antioxidant synergy, *via* mitochondrial FA-mediated uncoupling, slightly decreased superoxide/H_2_O_2_ production [37].

Both pathological and preventive roles of mitochondrial phospholipases-A_2_ iPLA_2_γ/PNPLA8 and iPLA_2_β/PNPLA9 have been reported. Both belong to the group-VI of PLA_2_s [48,49], ascribed to the cytosolic Ca^2+^-independent iPLA_2_s, patatin-like phospholipase domain-containing proteins (PNPLAs). Besides the typical release of unsaturated FAs by cleaving the *sn*-2 ester bond of membrane phospholipids, PNPLAs also cleave saturated FAs at the *sn*-1 ester bond [37,[48], [49], [50], [51]]. iPLA_2_s were previously indicated to participate in fuel sensing in pancreatic β-cells [52]. iPLA_2_γ knockdown promoted increases in membrane peroxidation and apoptosis induced by cytokines and pro-oxidants [53].

Previously, iPLA_2_γ/PNPLA8 knockout (KO) mice were reported to have impaired mitochondrial function associated with growth retardation, cold intolerance, and increased mortality due to aortic stress, all causing decreased myocardial function and respiration [54]. They exhibited severe impairment in skeletal muscle mitochondrial FA β-oxidation [[55], [56], [57]] and were resistant to Western diet-induced elevations in body weight, increases in glucose and insulin; but did not exhibit insulin resistance with concomitant impaired glucose tolerance, adiposity, and increased circulating cholesterol levels [57].

In this work, we demonstrate the existence of a redox signal originating from mitochondria and reaching the plasma membrane upon FA β-oxidation, which enables FASIS at low (insulin-non-stimulating) glucose. We demonstrated the above-described iPLA_2_γ-amplified FASIS mechanism *in vivo*, using in-house created PNPLA8-KO (iPLA_2_γKO) mice with no apparent impairment in β-oxidation. We also clarified these mechanisms for FASIS *in vivo*. Using isolated PIs, we show that both FASIS components depend on redox signaling, the metabolic component strictly on the mitochondria-to-plasma membrane redox signaling, while the GPR40-receptoric component predominantly on mitochondrial FAs. These FAs are cleaved by iPLA_2_γ, when activated by intra-mitochondrial redox signaling. Both redox signals have a common origin from FA β-oxidation.

## 2 Materials and methods

### 2.1 Chemicals and other materials

see Supplementary Table 1.

### 2.2 Creation of PNPLA8 knockout mice

Experiments were approved (Institute of Molecular Genetics committee), complying with the 2010/63/EU directive, NIH Publication No.85–23 (revised 1996) and the ARRIVE guidelines. The PNPLA8 knockout (KO) mice were generated using transcription activator-like effector nucleases (TALENs) [58], as previously described [59] (Fig. S1). The wild-type (wt) mice used were backcrossed >10 generations into the PNPLA8-knockout mice background.

### 2.3 Pancreatic islet perifusion and assays

PI isolation, yielding 100–200 islets *per* mouse, and perifusion was performed as described elsewhere [36]. 100–200 islets were placed into a column with an attached flow adaptor (1**×**7 cm Econo-Column, Bio-Rad, Hercules, CA), and immobilized with Bio-Gel P4 (Bio-Rad). PIs were washed for 60 min using a continual flow of Krebs-Ringer HEPES (KRH) buffer with 2.5 mM glucose: 135 mM NaCl, 3.6 mM KCl, 10 mM HEPES, 0.5 mM MgCl_2_, 1.5 mM CaCl_2_, 0.5 NaH_2_PO_4_, 0.2 % bovine serum albumin (30 μM fatty acid-free BSA), pH 7.4. When required, other tested agents were present 10 min before the stimulation of insulin secretion and then during the entire course of the assay. Perifusates were collected at rates of 0.5 ± 0.1 ml/min. Insulin was quantified using a Mouse-Insulin-High-Sensitivity-ELISA kit (BioVendor, Brno, Czech Republic). Islets were lysed and their DNA content was quantified using the PicoGreen Assay (ThermoFisher). AUCs were calculated for the FASIS 1st phase (0–16 min) and 2nd phase (18–50 min), while the minimum rates were subtracted as a background (alternatively a background with no PA was subtracted), and the results were calculated as a percentage relative to averages for wt control PIs with PA but without agents.

To detect extracellular H_2_O_2_ release, 10 μM Amplex-UltraRed and 5 IU of horseradish peroxidase were added *per* ml of perifusate, *i.e*. after collecting it from the column. Fluorescence intensity was detected using excitation of 572 nm and emission of 580 nm on an RF5301 PC spectrofluorometer (Shimadzu) at 37 °C and calibrated for each experimental condition by sequential additions of H_2_O_2_ aliquots. Measurements of oxygen consumption rates were performed with the Seahorse Extracellular Flux Analyzer XF24.

### 2.4 Preparation of fatty acid/BSA solutions

To prepare fatty acid (FA)/BSA solutions for experiments using islet perifusion or insulinoma INS-1E cell incubation in wells, selected FAs were pre-conjugated with BSA directly in the assay stimulation medium. Typically, FAs were dissolved in 50 % ethanol at 55 °C (stearate at 70 °C) and added to an assay medium containing 0.2 % (30 μM) BSA heated to 37 °C. The FAs were allowed to pre-conjugate with BSA under continuous mixing at 37 °C for 1 h prior to the experiment. Sodium salts of FAs were used when available. Typically, the ratios of long-chain FA:BSA ranged between 1.6 and 2.3. For kinetic experiments in a cuvette, where changing of solutions was not possible, selected FAs heated to 50 °C (stearate to 70 °C) were added directly during the run to the assay medium containing 0.2 % BSA. For the Seahorse experiments, the pre-conjugation of sodium palmitate to BSA at a 6:1 ratio was performed following the Seahorse protocol (“Preparation of Bovine Serum Albumin (BSA)-Conjugated Palmitate”, retrieved from: http://www.agilent.com/en-us/products/cell-analysis(seahorse)/seahorse-xfconsumables/kits-reagents-media/seahorse-xf-palmitatebsa-fao-substrate.); and the final ratio of palmitate/BSA during the assay was set to 1.6 by adjusting the concentration of BSA in the assay medium.

### 2.5 ATP quantification

Cells or PIs were lysed by boiling in 100 mM Tris-Cl, 4 mM EDTA, pH 7.75, for 2 min, while bioluminescence was determined using an ATP Bioluminescence Assay kit HSII (Roche, Basel, Switzerland) and a Luminometer Synergy HT microplate reader (Bio-TEK/Agilent, Winooski, VT, USA).

### 2.6 Experiments with INS-1E cells

Rat insulinoma INS-1E cells, were purchased from AddexBio (San Diego, CA; No. C0018009; RRID:CVCL_0351) and were cultivated with 11 mM glucose in RPMI 1640 medium with l-glutamine, supplemented with 10 mM HEPES, 1 mM pyruvate, 5 % (v/v) fetal calf serum, 50 μmol/l mercaptoethanol, 50 IU/ml penicillin, and 50 μg/ml streptomycin. Cells were seeded at 0.2 ·10^6^ cells/well in poly-l-lysine-coated 12-well plates one day before transfections, three days before experiments. Cells were preincubated/washed twice for 15 min with 3 mM glucose in KRH with 0.1 % or 0.2 % BSA. Insulin release was assayed using a Rat-Insulin-High-Sensitivity-ELISA kit (BioVendor, Brno, Czech Republic). For catalase overexpression (cf [36]) pZeoSV2(+) vector bearing human catalase sequence (a kind gift from C. Glorieux and Prof. J.B. Verrax, Universite’ Catholique de Louvain, Belgium) was transfected using Lipofectamine 2000 (2.5 ml/mg DNA; ThermoFisher Scientific). Similar transfection was used [36] for HyPer-7 overexpression, performed using a corresponding vector (pCS2+HyPer7, Addgene). MitoSOX Red, thalium, and other assays were conducted as described elsewhere [36,37,60]. CPT1 silencing employed the available Silencer Select siRNA (ThermoFisher, ID: s130674 Cpt1a). To assay the extracellular H_2_O_2_ release, 10 μM Amplex-Red plus 5 IU·ml^−1^ HRP was added to 10^6^ cells in a cuvette of an RF5301 PC spectrofluorometer (Shimadzu) at 37 °C. Fluorescence intensity was detected using excitation of 572 nm and emission of 580 nm. Calibrations were performed with 10 nmol H_2_O_2_ aliquots. Alternatively, 5 μM 2′,7′-dichlorodihydrofluorescein diacetate (DCF) was used to monitor the cytosolic H_2_O_2_ release.

### 2.7 GCaMP6 assay for Ca^2+^ oscillations in INS-1E cells

pGP-CMV-GCaMP6s vectors (Plasmid #40755), expressing the slow-responding but more sensitive GCaMP6 fluorescent Ca^2+^indicator [61], were purchased from Addgene. INS-1E cells were transfected using Lipofectamine™ 2000 (ThermoFisher) for 48 h. Before each experiment, cells were preincubated for 60 min (two washes of 30 min each) with 3 mM glucose in the same KRH buffer with 0.1 % BSA, in which monitoring was conducted. A Leica TCS SP8 confocal microscope was employed for the time-lapsed recording of integral fluorescence intensity within the individual cells ROI with excitation at 480 and emission at 510 nm. For every second, integral fluorescence intensities *F* [Ca]_c_ (t_i_) were collected from a widefield image of each responding cell, and data were plotted as a time course. Typically, 10 min recordings were set; in each recording either palmitic acid with or without the studied agents or increasing levels of glucose were added, starting from 3 mM, and at the end 30 mM KCl was added. The numerical derivative was calculated to ascertain peaks of oscillations, so that a peak at time *t*_i_ occurred for the derivative of *F* [Ca]_c_ (*t*_i_) being zero at time *t*_i_. Peaks of oscillations in each trail were sorted by intensities into ranges scaled by 1/10 of maximum intensity (deciles or 10-percentiles) and histograms were plotted for each recording. Histograms for PA-induced *F* [Ca]_c_ (*t*_i_) oscillations were unchanged with 0.5 μM carnitine included.

### 2.8 Unbiased lipidomics and profiling of carnitines in INS-1E cells

Lipidomics data were collected through the LIMeX platforms as described elsewhere [62], while INS-1E cell samples were collected before and 60 min after palmitic acid addition (15 μM with 15 μM BSA). For the analysis of ^13^C-labeled carnitine species in INS-1E cells, an optimized LC-MS based method was used as described elsewhere [63]. Briefly, cell pellets were washed with 137 mM NaCl, 2.7 mM KCl and resuspended in 250 μl of extraction buffer containing 40 % acetonitrile, 40 % methanol, 20 % water, and 0.1 M formic acid with 2 μg/ml indole-2,4,5,6,7-d5-3-acetic acid (CDN Isotopes, Pointe-Claire Quebec, Canada) as internal standard, and vortexed. Samples were centrifuged at 16,000×*g* for 20 min and 200 μl of supernatants were evaporated and suspended in 60 μl of 10 % acetonitrile containing 1 μg/ml d4-tyrosine (CDN Isotopes), 0.5 μg/ml Val-Tyr-Val (Merck).

The analysis was performed using a Dionex Ultimate 3000RS liquid chromatography system in conjunction with an Orbitrap Fusion mass spectrometer (Thermo Scientific). The separation was done on an XBridge Premier BEH C18 column (150 × 2.1 mm, 2.5 μm) with a VanGuard pre-column (2.1 × 5 mm, 2.5 μm). The mobile phase consisted of A (10 mM ammonium formate in water, 0.1 % formic acid) and B (99.8 % methanol) at a flow rate of 0.3 ml/min. The sample injection volume was 5 μl. The detector was set to Orbitrap Fusion mass spectrometer using ESI source in positive mode. Fragmentation spectra were collected using Thermo Scientific™ AcquireX Deep Scan acquisition on unlabeled sample. Due to impractical long-chain CoA analysis, carnitines were analyzed instead 60 min after U–^13^C-palmitic acid addition (50 μM with 30 μM BSA). During the 1st cycle of β-oxidation, eight M+2 acetyl-CoAs is released (two ^13^C), while carnitine O-acetyltransferase (CRAT) may convert them to the detected M+2 acetyl-carnitines. Similarly, in the last but one step of β-oxidation, M+4 butyryl-CoAs is released and converted to M+4 butyryl-carnitine (four ^13^C). Both ^13^C-labeled species thus indicate the ongoing β-oxidation (Supplementary material, Scheme S1).

### 2.9 Tests on mice

FASIS was estimated as follows: palmitic acid (PA, stock 1 mM at 0.17 mM BSA; 10 μg/g body weight) or 7.5 μg/g Intralipid or 10 μmol kg^−1^ Agonist II was administered by intraperitoneal (i.p.) injections into overnight (14-hr) fasted mice of mixed sex. Alternatively, oral administration was made. When indicated, r-bromoenol-lactone (r-BEL, 1 μg/g) was i.p. injected on the preceding four days. Blood was sampled at 2–3 time points from the eye plexus blood vessel for each mouse. Insulin and c-peptide were detected using an ELISA kit (Mercodia, Uppsala, Sweden; and ThermoFisher, respectively). Averaging the data of *N* = 10–40 mice enabled the construction of time-dependencies of insulin release [36]. Blood [glucose] was determined in samples with a glucometer (Roche, Basel, Switzerland). GSIS was tested after the i.p. injection or oral administration of glucose (1 mg/g body weight; ∼111 μmol glucose *per* mouse). Insulin resistance was measured as described elsewhere [36].

### 2.10 Statistical analysis

Biological (*N;* number of mice, PI isolations, cell passages; or for GCaMP6 confocal microscopic assay, number of single-cell confocal images) and experimental (*n*) replicates are listed. Single tail ANOVA (T-test for two quantities) used pairwise multiple comparisons (Tukey's test) on the pre-validated data using SigmaStat 3.1 (Systat-Software, San Jose, CA).

## 3 Results

### 3.1 Metabolic vs. receptoric FASIS involves minor vs. predominant participation of iPLA_2_γ

**Fig. 1:**
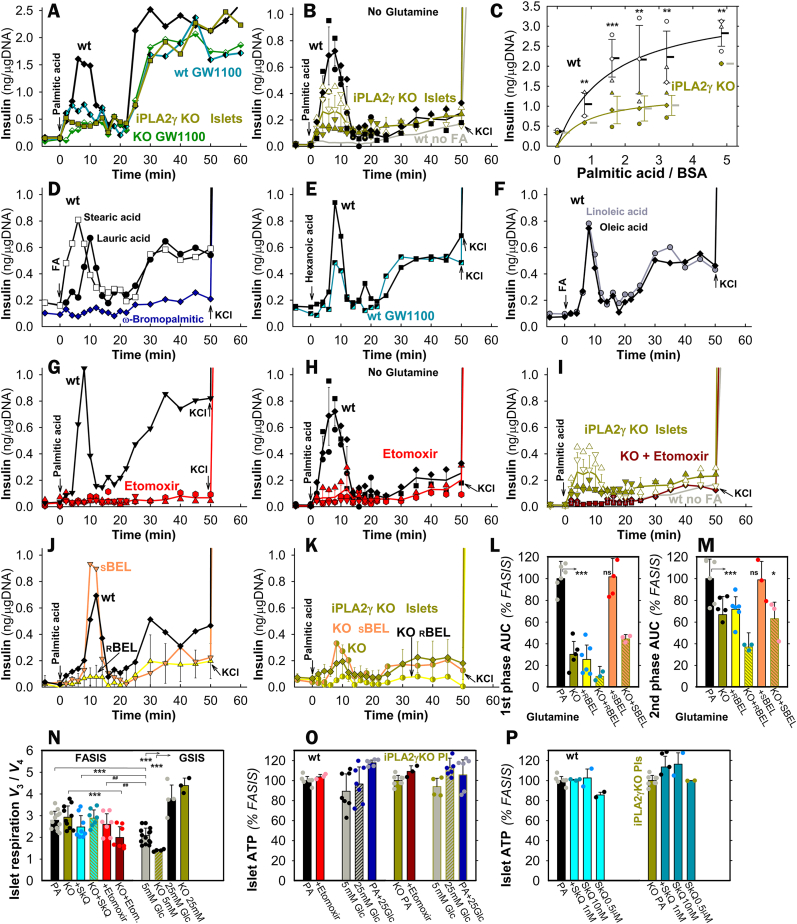
Fatty acid-stimulated insulin secretion (FASIS) in perifused isolated murine pancreatic islets – A,**B**,**D**–**K**) Instantaneous yields of insulin secretion are plotted *vs*. time. They represent the accumulated amount of insulin over each previous 2 min (5 min after 25 min). Hence, “ng/μgDNA” can be easily transferred into rates of insulin secretion in units of 0.5 ng min^−1^·μgDNA^−1^ up to 22 min and 0.2 ng min^−1^ ·μgDNA^−1^ after 25 min of perifusion (data after recalculation, see Fig. S2A). 100 PIs of backcrossed wt mice (*black, open, gray, or black/cyan semi-filled symbols*) and iPLA_2_γKO mice (*green, dark-green, green/yellow or green/orange semi-filled symbols*) were initially perifused with the KRH medium containing 5.5 mM glucose. Arrows indicate the moment from which 2.55 nM free palmitic acid (PA) or other FAs were included (all in 30 μM, *i.e*. 0.2 % BSA: total PA and linoleic acid were 50 μM; stearic 65 μM, oleic 70 μM, lauric 90 μM, hexanoic 180 μM, and ω-bromopalmitic 50 μM). For perifusion without FAs, see *gray trace* (“*wt no FA”* in **B**). Representative perifusions are shown (otherwise, *N* = 3–7 for each type). After 50 min (60 min in **A**), 30 mM KCl was added (arrows). Panel **C** shows the accumulated amount of insulin secreted at 0–12 min (derived from areas under curve, AUCs), while data are plotted as the PA dose-response at constant BSA (30 μM). AUCs relative to PA are summarized in Figs. S2E and F (2–16 min for the 1st phase, 18–50 min for the 2nd phase). For other AUCs and AUCs of iPLA_2_γKO PIs see Fig. 2A and B below and Fig. 2 legend. Additional agents were also supplied over the entire time course of perifusion when indicated, such as 1 μM GW1100 (*black/cyan* or *dark green/white semi-filled symbols*; perifused 10 min before FAs) **(A**,**E)**; 2.5 μM etomoxir (*red*/dark red) **(G**–**I)**; and 2.5 μM r-BEL (*yellow*) or 2.5 μM s-BEL (*orange*) (**J**,**K**). The latter assessed stereospecific FASIS inhibition by r-BEL, as also expressed by AUCs (**L**,**M**). **N**–**P)**: **Respiration and ATP levels in pancreatic islets – N**) The respiration of PIs (*N* = 5; *n* = 3–15) was assayed on a SeahorseXF analyzer (exemplified in Fig. S4) and ratios of rates obtained without (*V*_3_) and with oligomycin (*V*_4_) are plotted for standard FASIS (“PA”) under conditions of Fig. 1A with the indicated agents and also without PA, but with indicated glucose (“Glc”) concentrations. **O**,**P**) ATP levels 60 min after the addition of PA, or PA plus denoted agents, are shown with and without indicated agents (*N* = 3; *n* = 3–7). In **N**–**P**), Etomoxir (*red*, *violet*) was 2.5 μM; SkQ1 10 nM (*cyan;* “*SkQ”*) (**O**) or as indicated (*cyan*) **(Q)**. In panels **C**,**L**–**P**, ANOVA is indicated: **p < 0.05; ***p < 0.001; and Student's T-test between two groups: ##*p* < 0.05.

Two phases of insulin secretion were clearly distinguished when 50 μM palmitic acid (PA; 2.55 nM free with 30 μM BSA) was supplied during the perifusion of wt pancreatic islets (PIs) with a KRH-medium containing 5.5 mM glucose (Fig. 1A–C; S2A). The 1st phase, peaking at ∼8 min, dropped down to almost the basal rates at ∼16 min. The 2nd phase then began spontaneously, lasting >40 min and having higher insulin secretion rates, when 2 mM glutamine was present (Fig. 1A *vs*. 1B; 1G *vs*. 1H). At doses allowing theoretically similar free FA concentrations with 30 μM BSA (Table S2), 65 μM stearic (Figs. 1D), 90 μM lauric (Figs. 1D), 180 μM hexanoic (Figs. 1E), 70 μM oleic, and 50 μM linoleic acids (Fig. 1F) also stimulated insulin release (Figs. S2E and F). A minimum insulin secretion by perifused PIs was observed with non-metabolizable ω-bromopalmitic acid (Fig. 1D; S2E,F).

**Fig. 2:**
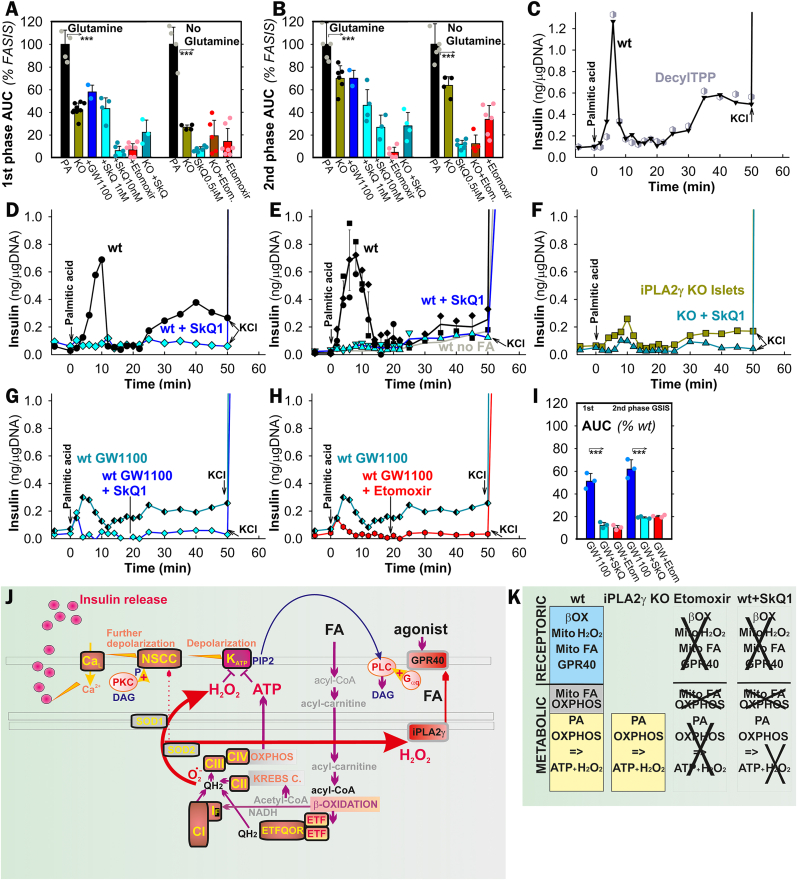
FASIS is prevented by mitochondria-targeted antioxidants SkQ1 **A**,**B)** To demonstrate the secreted insulin amounts, AUCs are quantified in ng/μg DNA for all measured perifusions under conditions of Fig. 1A plus those with 1 and 10 nM SkQ1 (*cyan, dark cyan;* “*SkQ”*), such as exemplified in traces of panels **D**–**F** with 10 nM SkQ1 (*cyan, dark cyan*) **(D**,**F)**, or 0.5 μM SkQ1 (*cyan*) **(E)**. The inactive SkQ1 mimics 10 nM DecylTPP was tested as illustrated in panel **(C)**; as well as FASIS inhibition with 10 nM SkQ1 and 2.5 μM etomoxir on the top of 1 μM GW1100 was assayed as shown in panels **G**,**H**, while AUCs (*N* = 3) are summarized in panel **I**. ANOVA between groups is indicated: **p < 0.05; ***p < 0.001. **(J)** Scheme of working hypothesis: **redox signaling from mitochondria to the plasma membrane and intra-mitochondrial redox signaling upon FASIS**. CI–CIV are respiratory chain complexes (a superoxide-forming flavin site is denoted I_F_); ETFQOR is ETF:ubiquinone-oxidoreductase; DAG stands for diacylglycerols; NSCC for nonspecific calcium channels, such as TRPM channels; „PIP2″, i.e. PIP_2_ is phosphatidylinositol 4,5-bisphosphate, required to be unbound from K_ATP_ to release its permanent opening. **K**) **Contribution of various components to insulin release upon FASIS**.

As exemplified with PA and hexanoic acid, both FASIS phases were incompletely suppressed by a GPR40-antagonist GW1100 (Fig. 1A–E; Fig. 2A and B). With PA, the 1st phases yielded insulin amounts (areas under curves, AUCs) approaching those of GSIS (25 mM glucose no PA; Figs. S2B–D); the 2nd-phase yield was slightly lower than for GSIS. Despite their GSIS 1st phases and initial responses to glibenclamide (Fig. S3A) being equal to the wt, iPLA_2_γKO-PIs with/without glutamine exhibited suppressed insulin secretion in the FASIS 1st phase by 60 %/70 %, respectively (Fig. 1A–C,I,K,L; Fig. 2A), with the 2nd phase being affected less (by 25 %/30 %; Fig. 1A–C,I,K,M; Fig. 2B *vs*. 2A), independently of GW1100 (Fig. 1A).

The GW1100-sensitive and insensitive portions could be ascribed to “receptoric” and “metabolic“ FASIS, (non-fuel and fuel), respectively. Since with PA, the former was negligible in iPLA_2_γKO-PIs, the cleaved mitochondrial FAs, but not the added palmitic acid, should dominantly activate GPR40 in wt-PIs. This should be similar to hexanoic acid (itself being partly interacting also with GPR41 and GPR43 [24,30]). FASIS with PA was also inhibited with r-bromoenol-lactone (r-BEL, not s-BEL), a stereo-selective inhibitor of iPLA_2_γ/PNPLA8 (Fig. 1J–M). Evidently, the absence of mitochondrial FAs substantially prevents receptoric FASIS [30] (Fig. 1J), which otherwise dominates the 1st phase; but partly also metabolic FASIS, in both phases (Fig. 1K), preventing cleavage and possible subsequent β-oxidation of mitochondrial FAs.

**Fig. 3:**
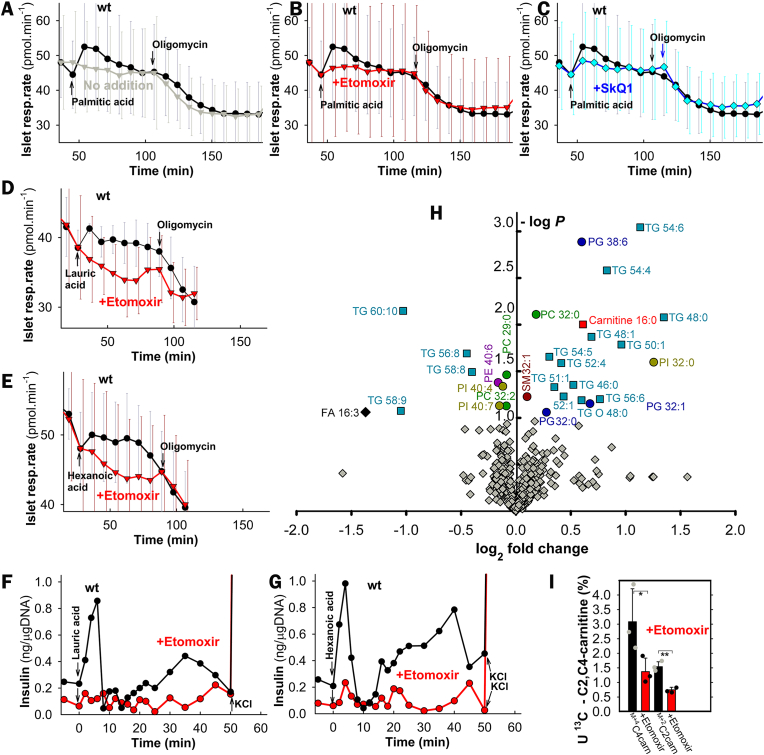
Evidence for increasing β-oxidation upon addition of various FAs to pancreatic islets A–**E) Respiration rates of pancreatic islets** (averaged from *N* = 5) without any addition (*gray*) and with added 50 μM palmitic acid (30 μM BSA) alone (**A**); or in the presence of 2.5 μM etomoxir (**B**) and 10 nM SkQ1 (**C**). When indicated, 5 μM oligomycin was added. Time window is selected to emphasize PA-induced respiration increase. For examples of complete timing, see Fig. S4 A-C,D,E**)** Exemplar islet respiration (otherwise *N* = 3) before and after the addition of 70 μM, lauric (**D**) and 90 μM, hexanoic acid (**E**) (both with 30 μM BSA) without and with 2.5 μM etomoxir. **G**,**H)** A complete etomoxir blockage of insulin secretion (FASIS) induced with lauric and hexanoic acid, under the same conditions as for panels **D** and **E**. **H) Unbiased lipidomics** of INS-1E cells – a Volcano plot shows statistically significant changes 60 min after the addition of 15 μM palmitic acid (15 μM BSA; *N* = 6). TG – triglyceride; PC – phosphatidylcholine; PE – phosphatidyletanolamine; PG – phosphatidylglycerol; PI – phosphatidylinositol; SM – sphingomyelin. **I) Existence of M** + **4**^**13**^**C-butyryl-carnitine** („C4carn”) and **of M** + **2**^**13**^**C-acetyl-carnitine** („C2carn”) in INS-1E cells 60 min after U–^13^C-PA addition (50 μM, 30 μM BSA; *n* = 3) indicates the ongoing β-oxidation. It was inhibited by 2.5 μM etomoxir. Data multiplied by 100 (i.e. expressed in %) represent calculated ratios of M+4/(M+0 + M+4) and M+2/(M+0 + M+2), respectively.

Etomoxir, an inhibitor of carnitine palmitoyltransferase 1 (CPT1, and hence of β-oxidation), completely blocked FASIS in wt-PIs (Fig. 1G and H; Fig. 2A,B,H,I; Fig. 3G–I), despite the largely preserved phosphorylating to non-phosphorylating respiration ratio (indicating the oxidative phosphorylation, OXPHOS, Fig. 1N; Fig. S4) and elevated ATP levels (Fig. 1O). This demonstrates that by itself having higher ATP levels is insufficient for insulin secretion. Palmitic acid increased islet respiration (Fig. 3A–C), similarly to other FAs (exemplified by lauric and hexanoic acid; Fig. 3D and E). This was prevented by etomoxir, complying with the complete etomoxir blockage of insulin secretion (i.e. FASIS; Fig. 3G and H). Since etomoxir prevented the FA-induced respiration increase, we can conclude that the respiration increase reflects FA β-oxidation. Direct evidence for the elevated FA β-oxidation in INS1-E cells was provided using an unbiased metabolomics, indicating enrichment of palmitoylcarnitine (Fig. 3F) and by surveying downstream products of U–^13^C-palmitate β-oxidation, namely M+2 acetyl-carnitine (i.e. two ^13^C) and M+4 butyryl-carnitine (i.e. four ^13^C), which were decreased by etomoxir (Fig. 3I).

In PIs with PA, with/without glutamine, etomoxir blocked the FASIS 1st phase by 93 %/86 % (2nd phase by 95 %/66 %), respectively. Both residual FASIS phases with PA in iPLA_2_γKO-PIs also ceased with etomoxir (Fig. 1I; Fig. 2A and B), but at diminished OXPHOS (Fig. 1N). The complete blockage with etomoxir stems from the absence of the initiating event, *i.e*. the PA-supplied β-oxidation-derived H_2_O_2_ release (see below), which activates the iPLA_2_γ-mediated cleavage of mitochondrial FAs [37] (Fig. 2J and K). With etomoxir, mitochondrial FAs are neither cleaved, nor activate GPR40. Therefore, also the receptoric FASIS is missing as well as the metabolic one, which is missing due to the absence of β-oxidation.

### 3.2 Requirement of redox signaling for FASIS

Unlike decyltriphenylphosphonium bromide (Decyl-TPP, Fig. 2C), the mitochondrial-matrix-targeted antioxidant SkQ1 (10-(6′-plastoquinonyl)decyltriphenylphosphonium) prevented both FASIS phases in wt-PIs (Fig. 2A,B,D,E) and iPLA_2_γKO-PIs (Fig. 2A,B,F), blocking them by ∼60 % at 1 nM; >90 % of AUC^1st^; (>70 % of AUC^2nd^) at 10–500 nM. PI's OXPHOS and ATP levels were virtually unaffected by 1–10 nM SkQ1 (Fig. 1N–P; in iPLA_2_γKO-PIs even by 500 nM SkQ1). Again, with SkQ1, we observe enough high ATP (Fig. 1P) and OXPHOS (Fig. 1N; Fig. S4), but no insulin secretion. Collectively, these data suggest the participation of mitochondrial redox signaling (Fig. 2J). Specifically, the SkQ1 inhibition of GW1100-suppressed FASIS (Fig. 2G–I) indicates mitochondria-to-plasma-membrane redox signaling for the net metabolic FASIS regime.

SkQ1 contains plastoquinone linked to decyltriphenylphosphonium and drains electrons from the respiratory chain Complex I, sites I_F_, I_Q_, and Complex III site III_Qo_, *i.e*. hypothetical candidate sources of superoxide (O_2_^•**–**^) upon FA β-oxidation [[64], [65], [66]], besides the ETF:ubiquinone-oxidoreductase (ETFQOR; ETF is the electron-transfer flavoprotein) [40,65]. By preventing excessive O_2_^•**–**^ formation in any of these sites, SkQ1 should disrupt redox signaling activating iPLA_2_γ and prevent metabolic FASIS triggering (disable K_ATP_ closing), notably its 2nd phase. Since S1QEL and S3QEL, suppressors of O_2_^•**–**^ specific for sites I_Q_ and III_Qo_, respectively [65,66], did not inhibit FASIS (Figs. S3B–G), some other sites should be relevant, such as I_F_.

**Fig. 4:**
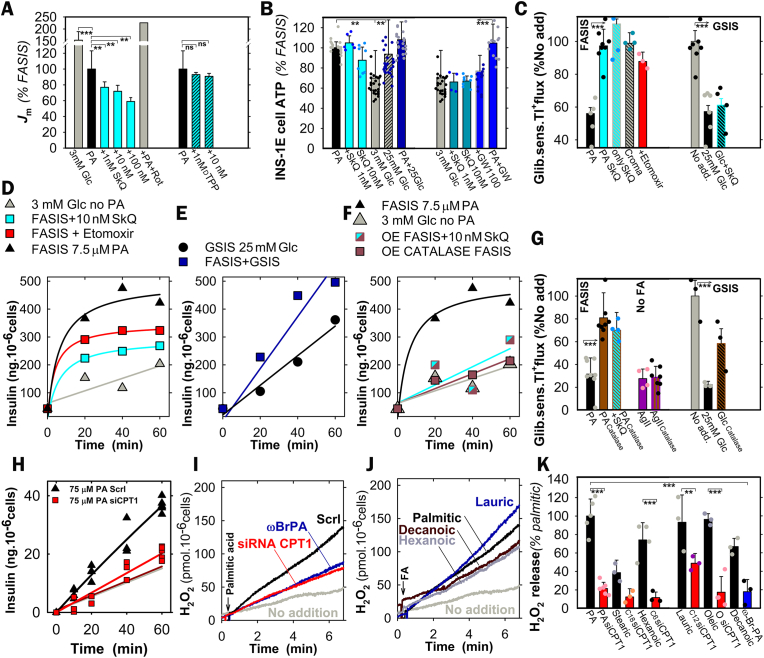
FASIS in INS-1E cells **A**) **Superoxide** (O_2_•^**–**^) **release** into the mitochondrial matrix, monitored under the confocal microscope in INS-1E cells with MitoSOX Red at standard 15 μM palmitic acid (1.3 nM free PA with 15 μM BSA). Relative rates *J*_m_ with agents were normalized to standard ones (”% FASIS”; *N* = 8, *n* = 12–15). When indicated (*n* = 3–8), 1 nM, 10 nM, and 100 nM SkQ1 blocked 23 % ± 7 % (*N* = 8), 28 % ± 8 % (*N* = 8), and 41 % ± 5 % (*N* = 3) of the average *J*_m_ rates of O_2_^•**–**^ release. Compare to the negligible effects of Decyl-TPP (“dTPP”; 1 and 10 nM) and the maximum effect of 20 μM rotenone (“Rot”). **B**) **ATP levels** established upon FASIS or GSIS after 60 min (conditions of panel A; *N* = 3; *n* = 8–20). **C**) **K**_**ATP**_**-channel closing status** in ensemble, taken as glibenclamide-sensitive rate of Tl ^+^ influx after 6th min of cell preincubations (*N* = 7; *n* = 3–7; glibenclamide was 100 μΜ) in KRH induced with 15 μM PA (15 μM BSA; 1.3 nM free PA) with no other agents (“PA”) or together with 10 nM SkQ1 (“PA SkQ”), 50 μM cromakalim (“Croma”), and 2.5 μM etomoxir. The effect of SkQ1 without PA was also tested (“only SkQ”). However, upon GSIS with SkQ1, K_ATP_ remained to be closed (panel **C**, right). **D**–**F) Insulin** secreted and accumulated over the indicated time in representative experiments (*N* = 3), but with only 1.5 μM BSA and 7.5 μM PA (21 nM free). This is compared to no addition (“No. add.“, *i.e*. 3 mM glucose; *gray triangles* in **D**,**F**). Etomoxir (*red squares*) was 2.5 μM; SkQ1 (cyan) was 10 nM. **E**) GSIS at 25 mM glucose is compared to simultaneous FASIS and GSIS (*dark blue*). **F**,**G**) **Cytosolic catalase overexpression – Insulin** (**F**; conditions see panel D) and **K**_**ATP**_ (**G**, 15 μM BSA; 15 μM PA, i.e. 1.4 nM free) were tested for INS-1E-cells transfected with a control empty-vector (*black*) *vs*. catalase vector (*brown*), the latter blocking FASIS **(F)** and **preventing** K_ATP_ closure of transfected cells (a thallium assay, **G**) (*N* = 3; *n* = 3–7). SkQ1 was 10 nM (*cyan/brown semifilled squares*); or no PA was present (*cyan* or *brown* in **G**). Tl ^+^ flux suppression upon GSIS is also indicated in panel **G**, plus the prevention of such suppression in catalase-expressing cells. **H**,**I**,**K**) **Carnitine palmitoyltransferase 1 silencing –** Insulin (**H**) and Amplex UltraRed monitoring of **H**_**2**_**O**_**2**_**release** to the INS-1E cell exterior (**I**,**K**) for cells transfected with siRNA bearing scrambled sequence (“Scrl”, *black*) and siRNA CPT1 (*red*). Insulin FASIS assay was conducted with 75 μM PA in 30 μM BSA (4.55 nM free PA). **J**,**K**) **H**_**2**_**O**_**2**_**release** to the INS-1E cell exterior for other FAs at 30 μM BSA: **J**) exemplar records for 50 μM PA, ωBrPA, and linoleic; 70 μM oleic; 90 μM decanoic, and lauric, plus 180 μM hexanoic acids (for free FA see Table S2). **K**) Comparison of net H_2_O_2_ release initial rates with subtracted rates of no FA addition.

FA β-oxidation provides potential enhancers of mitochondrial O_2_^•**–**^ generation, *i.e*. the surplus input of acetyl-CoA, NADH, and ubiquinol (QH_2_; by ETFQOR) [40,65], despite antioxidant UCP2-iPLA_2_γ synergy, which somewhat decreases O_2_^•**–**^ generation directed to the matrix [37] (Fig. 4A). MitoSOX-Red O_2_^•**–**^ monitoring in INS-1E cells confirmed that unlike Decyl-TPP, 1–100 nM SkQ1 acted as an instant antioxidant, blocking 23–41 % of the O_2_^•**–**^ release rates into the mitochondrial matrix at 15 μM PA (15 μM BSA, 1.3 nM free PA) (Fig. 4A). This correlated with the inhibition of FASIS (Fig. 4D) and open K_ATP_ channels, assayed *via* Tl^+^ fluxes (Fig. 4C). PA-induced ATP elevations upon cell FASIS (approximately equal to those of GSIS) were preserved at 1–10 nM SkQ (Fig. 4B) and unaffected also by GW1100. Any interpretation that SkQ1 acts *via* decreasing OXPHOS, and hence declining ATP, is excluded by the fact that GSIS [36], including K_ATP_ closing (Fig. 4C) is unaffected by SkQ1. Still, FASIS is prevented by SkQ1, which keeps K_ATP_ open (Fig. 4C).

FASIS in INS-1E cells with 21 nM free PA (7.5 μM PA, 1.5 μM BSA) exhibited a non-linear initial insulin release accumulation (Fig. 4D), faster than GSIS, while 25 mM glucose slightly facilitated FASIS (Fig. 4E) [37]. The role of FA β-oxidation in FASIS of intact INS-1E cells was also evidenced by ∼90 % prevention of FASIS in cells silenced for CPT1 (Fig. 4H; 4.6 nM free PA at 75 μM PA with 30 μM BSA), whereas no prevention existed for GSIS (Fig. S5). Thus INS-1E cells deficient for a key β-oxidation enzyme CPT1 did not secrete insulin, but also released H_2_O_2_ (*vide infra*) in much slower rates after FA addition, for all tested FAs (Fig. 4 I–K).

Also, results with overexpressed cytosolic catalase in INS-1E cells supported the existence of redox signaling from mitochondria to the cytosol. Cytosolic catalase, which degrades H_2_O_2_, blocked FASIS with PA (Fig. 4F), while simultaneously preventing the K_ATP_ closure, similarly as it did for GSIS (Fig. 4G). Thus, with insufficient redox signaling, K_ATP_-channels cannot be closed [36].

### 3.3 Direct monitoring of redox signaling in pancreatic islets and INS-1E cells upon FASIS

**Fig. 5:**
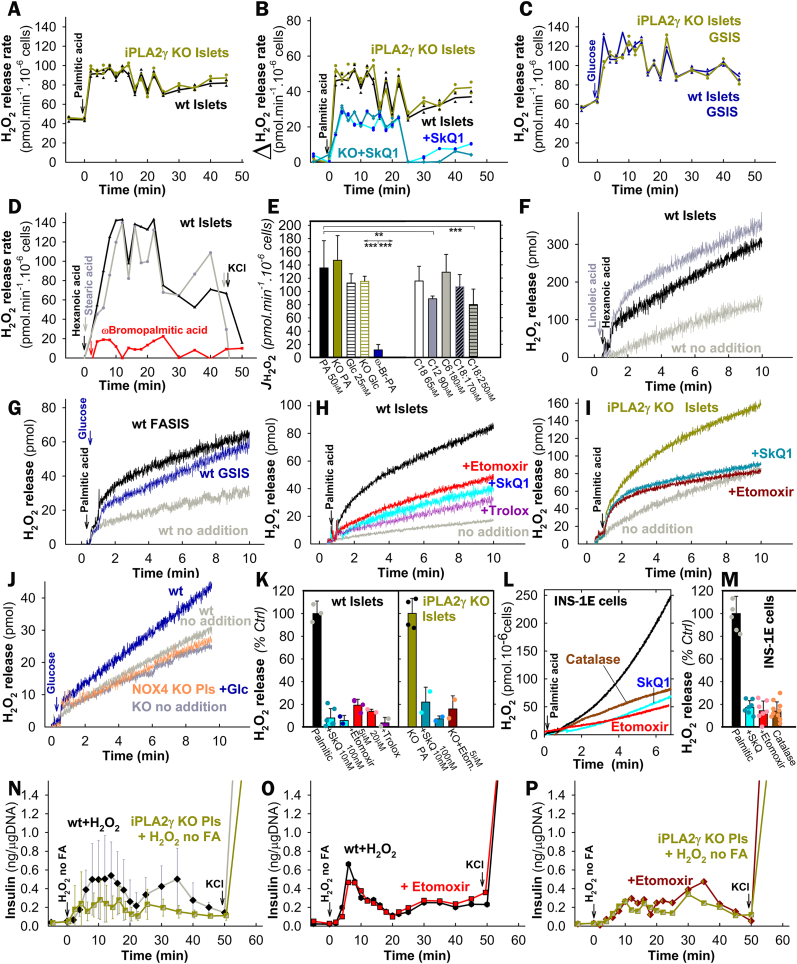
Extracellular monitoring of redox signaling upon FASIS *vs*. GSIS and H_2_O_2_ “rescue” of insulin release without FAs A–**M)** Extracellular H_2_O_2_ quantification with Amplex UltraRed and HRP in PIs (**A**–**K**) and INS-1E cells (**L**,**M**). Assays were calibrated with 2 μl aliquots of 10 μM H_2_O_2_. **A**–**E)** 10 μM Amplex UltraRed plus 5U/ml HRP were added to perifusate samples after their collection from the column containing 200–280 islets; representative data (otherwise *N* = 7) indicate rates of duplicates at each time point upon FASIS with 0.2 % (30 μM) BSA and with 50 μM PA (**A**,**B**), 65 μM stearic, 180 μM hexanoic, and 50 μM ω-bromopalmitic acid (**D**,**E**); 70 μM oleic, 90 μM lauric and 50 μM linoleic acid (**E**); or GSIS with 25 mM glucose (**C**) for wt or iPLA_2_γKO-PIs (*green*); or with 10 nM SkQ1 (*cyan/dark cyan*). For free FA calculations, see Table S2. **F**–**K)** „Static” incubations of >100 islets in a cuvette with 10 μM Amplex UltraRed plus 5 U/ml HRP. Representative data (**F**–**J**; summary for various FA doses see Fig. S6) indicate the accumulated H_2_O_2_ amount upon FASIS or GSIS (25 mM glucose) for wt *vs*. iPLA_2_γKO-PIs, and upon GSIS for NOX4KO-PIs [36] (panel **J**, *orange*) *vs*. their own backcrossed controls [36] (“wt”). Effects of selected agents are compared in panel **K** (*N* = 3–5). When indicated, 10 nM SkQ1 (*cyan/dark cyan*), 5 μM etomoxir (*red/dark red*) or 100 μM Trolox (*violet*) were present (higher concentrations see panel **K**, where inhibition of net initial rates was calculated with subtracted rates of no addition). **L**,**M**) **INS-1E cells:** representative traces (**L**) and inhibitory effects after 1-hr pre-incubation (**M**; 2.5 μM etomoxir; *N* = 5; *n* = 5–10) for FASIS with 15 μM PA (1.4 nM free) in 0.1 % (15.5 μM) BSA. For overexpression of the cytosolic catalase (*brown*), rates with subtracted no addition rates were normalized to cells transfected with an empty vector (**M**). **N**–**P) Rescue of insulin secretion at low 5.5 mM glucose without FA by 100 μM H**_**2**_**O**_**2**_, *i.e*. representative perifusions (otherwise *N* = 3–6), mimicking insulin secretion by the external H_2_O_2_ present during perifusion of wt (*black*) and iPLA_2_γKO-PIs (*green*) in the absence and presence of 2.5 μM etomoxir in wt (*red*) (**O**) and iPLA_2_γKO-PIs (*dark red*) (**P**). For a summary of secreted insulin amounts (AUCs), see Fig. S7H.

Direct monitoring of redox signaling upon FASIS was demonstrated with Amplex UltraRed [66] plus horseradish peroxidase (HRP), quantifying H_2_O_2_ in perifusates, when PIs remained in a column (Fig. 5A–E). At low (5.5 mM) glucose, a somewhat constant rate of H_2_O_2_ release into the extracellular space within islets of 40–60 pmol min^−1^·10^−6^ PI-cells was observed (considering 1500 cells *per* a single islet; *N* = 5 of biological replicates, *n* = 10; Fig. 5A–C). With the added 50 μM palmitic acid (2.55 nM free with 30 μM BSA), an instant surplus of H_2_O_2_ release quickly saturated into a steady state rate of 136 ± 40 pmol min^−1^·10^−6^ PI-cells (e.g. Fig. 5A; otherwise, *N* = 7, *n* = 40 time points). This indicates the ability of the redox signal to reach the plasma membrane upon FASIS. Similar H_2_O_2_ release into the extracellular space was found with stearic (Fig. 5D), lauric, hexanoic (Fig. 5D), oleic, and linoleic acid, but not with ω-bromopalmitic acid (Fig. 5E).

Identical H_2_O_2_ release rates as in wt PIs were obtained with the same (50 μM; 2.55 nM free) PA dose in iPLA_2_γKO-PIs (147 ± 37 pmol min^−1^·10^−6^ PI-cells; *N* = 4, *n* = 18 time points; Fig. 5A,B,E); and similarly upon GSIS, for which NOX4 is an H_2_O_2_ source [36] (after 25 mM glucose: 113 ± 14 *vs*.115 ± 14 pmol min^−1^ ·10^−6^ PI-cells in wt *vs*. iPLA_2_γKO-PIs, respectively; *N* = 3; Fig. 5C–E).

An elevated accumulating extracellular H_2_O_2_ release upon FASIS and GSIS was also observed when Amplex UltraRed plus HRP were present in the cuvette with PIs (Fig. 5F–K; Fig. S7) or INS-1E cells (Fig. 4I–K; 5L,M), where it was blocked with overexpressed cytosolic catalase. Depending on PI number and FA doses, the ±FA differential rates saturated to similar relative rates normalized to rates with PA (Fig. 5F–J; S6A). With PA these ± FA differential rates saturated to 72 ± 38 (52 ± 13 in KO) pmol·min^−1^·10^−6^ PI-cells.

In INS-1E cells at 3 mM glucose before FAs, estimated rates of 6.6 ± 3 pmol H_2_O_2_·min^−1^·10^−6^ cells corresponded to 0.47 % ± 0.2 % of oxygen converted to superoxide, that was dismuted to H_2_O_2_ reaching the cell exterior at the given respiration rates (Fig. S7A) [37]. With PA (Fig. 4I and J; Fig. 5L and M), the estimated differential rates of 46 ± 6 pmol min^−1^·10^−6^ of INS-1E cells (*N* = 5) corresponded to 3 % ± 0.4 % of oxygen converted to superoxide/H_2_O_2_.

NOX4 was a primary H_2_O_2_ source for GSIS, as evidenced in NOX4 null KO PIs [36], where the surplus rate ceased after glucose addition (Fig. 5J). Note a zero surplus H_2_O_2_ release with ω-bromopalmitic acid (Fig. 4I–K; 5D,E) and Agonist II (Figs. S7B–D).

Besides being diminished by CPT1 silencing (Fig. 4I–K), H_2_O_2_ release to the cell/PI exterior upon FASIS was inhibited by 5–20 μM etomoxir (Fig. 5H,I,K–M) and by 10–100 nM SkQ1 (Fig. 5B,H,I,K–M); as well as by 100 μM Trolox (6-hydroxy-2,5,7,8-tetramethyl chroman-2-carboxylic acid), a water-soluble analog of vitamin E (Fig. 5H,K). Also, monitoring of cytosolic ROS downstream of H_2_O_2_, using 2′,7′-dichlorodihydrofluorescein diacetate (DCF) [67] in INS-1E cells, indicated redox signaling (Figs. S7B and C), as well as did HyPer-7 H_2_O_2_ monitoring [[68], [69], [70]] (Fig. S7E).

### 3.4 Mimicking insulin secretion responses with external H_2_O_2_

Without the added FA, a substantial insulin release was induced with external 100 μM H_2_O_2_ at 5.5 mM glucose [36] and was higher in wt-PIs *vs*. paired iPLA_2_γKO-PIs (Fig. 5N–P; S7F–H**)**, indicating possible amplification by mitochondrial FAs in wt-PIs. So, the predominantly „metabolic” 2nd phase was relatively low in iPLA_2_γKO-PIs. Note that this is probably modestly amplified in wt-PIs by cleaved mitochondrial FAs. Nevertheless, the external H_2_O_2_ triggered insulin directly, bypassing the FA β-oxidation redox source. This set-up does not allow etomoxir to inhibit insulin secretion (Fig. 5O and P; S7H). These results also exclude any non-specific effect of etomoxir in the used doses, which could be theoretically involved in experiments of Fig. 1G–I.

### 3.5 Mimicking GPR40 responses with non-metabolizable agonists

**Fig. 6:**
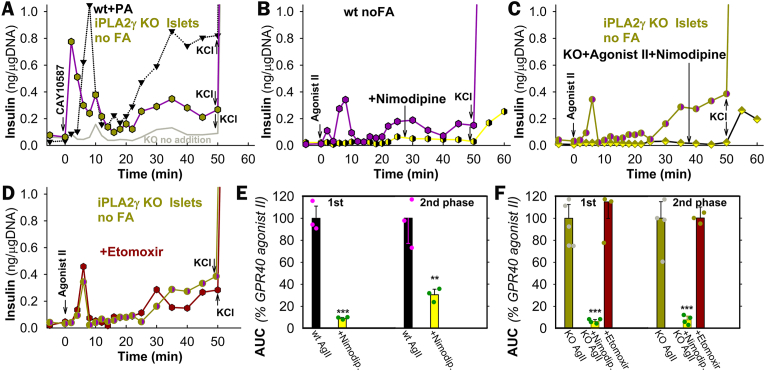
Insulin secretion responses to non-metabolizable GPR40 agonists **A**–**D)** Representative perifusions (number of repeats *N* = 3–5, see panels E,F), demonstrating the stimulation of insulin secretion at 5.5 mM glucose with GPR40-agonists CAY10587 (10 μM, *black edge* symbols) and Agonist II (1 μM, *violet*) in wt (**B**) and iPLA_2_γKO-PIs (*green, green edge*) (**A**,**C**,**D**) in the absence and presence of 5 μM nimodipine (*yellow*) (**B**,**C**), and 2.5 μM etomoxir (*dark red*) (**D**). Panels (**E**,**F**) summarize the respective yielded AUCs relative to Agonist-II assay with no other agents.

Surprisingly, at low resting glucose (5.5 mM for PIs) without any FA, an instantaneous (2nd and 8–10th minute biphasic) insulin release was stimulated by non-metabolizable GPR40 agonists CAY10587 and Agonist II (3-(4-((2,6-dichloropyridin-4-yl)ethynyl)phenyl)propanoate) in wt-PIs and iPLA_2_γKO-PIs (Fig. 6A,B,C). The „2nd phase” began at 20 min (AUC^2nd^ 60 ± 30 %/100 ± 30 % of FASIS in wt/iPLA_2_γKO-PIs). In accordance with the non-metabolizable character of Agonist II, both phases were insensitive to etomoxir (Fig. 6D–F), but were blocked entirely by nimodipine (Fig. 6B,C,E,F). The K_ATP_-dependent triggering upon Agonist–II–induced insulin secretion was suggested by the equally closed K_ATP_ population in INS-1E cells as established upon FASIS but insensitive to catalase overexpression (Fig. 4G).

### 3.6 FASIS dependence on Ca_L_-channel opening

**Fig. 7:**
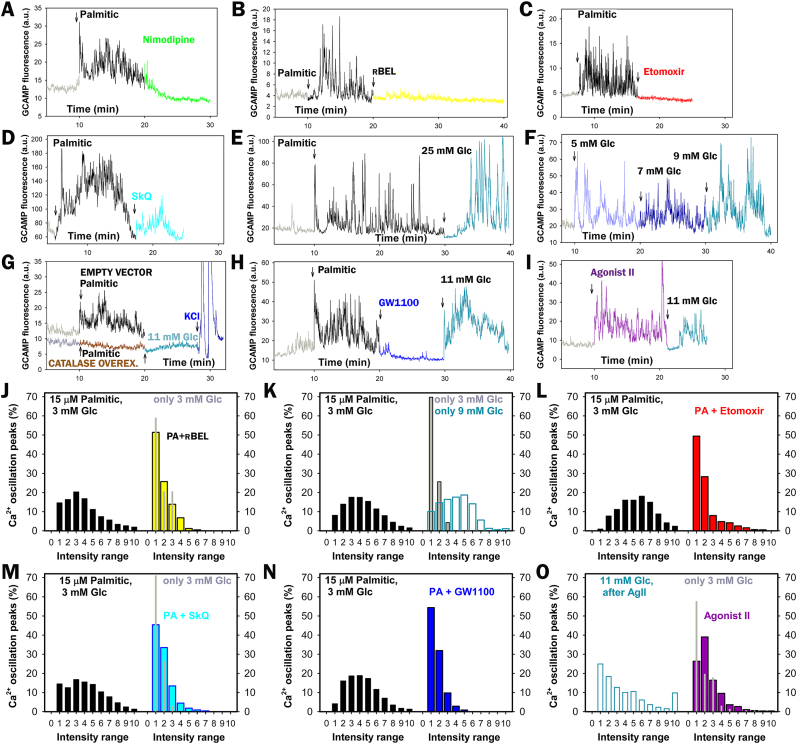
Dependence of FASIS on Ca_V_ opening – FASIS in INS-1E-cells was stimulated with 15 μM PA (15 μM i.e. 0.1 % BSA, 1.4 nM free PA; **A**–**E**,**G**,**H**) in typical single-cell recordings with additions of agents or glucose as indicated. **(A**–**I)** GCaMP6 fluorescence intensity records are shown for typical single-cell confocal monitoring of cytosolic Ca^2+^-oscillations [*Ca*^2+^]_c_(*t*) in KRH medium containing 3 mM glucose and induced with PA (**A**–**E**,**G**,**H**) or 1 μM Agonist II (**I**). Additions of 15 μM PA are indicated by arrows, as well as additions of 5 μM nimodipine (**A**), 2 μM r-BEL (**B**), 2.5 μM etomoxir (**C**); 10 nM SkQ1 (**D**), and 2 μM GW1000 (**H**). In panel **E**), glucose was adjusted to 25 mM after 30 min to probe FASIS at high glucose. In panel **F**), no PA was added, but step-wise adjustments of glucose to 5, 7, and 9 mM were performed, just probing for GSIS. Panel **G**) shows the absence of both FASIS and GSIS (at 11 mM glucose) in INS-1E overexpressing catalase (*bottom plot*) as compared to cells expressing the empty vector (*top plot*). 30 mM KCl was added at the end. Panels **(J**–**O)** show the peak analyses of Ca^2+^-oscillations – *N* = number of records in individual cells with 15 μM PA (1 μM Agonist II in **O**) are indicated, as well as the number of records with an inhibitor/agent *N*_inh_ that they were compared to (*N*/*N*_inh_): **J**) 8/8; **K**) 51 (8000 peaks); 23 for 3 mM glucose and 10 for 9 mM glucose; **L**) 6/6; **M**) 13/12; **N**) 20/20; **O**) 5; 5 for 11 mM glucose. Note that all available FASIS data are summarized in the leftmost histogram of panel **K**).

Nimodipine-sensitive FASIS depends on intermittent Ca_L_-opening, which was monitored using GCaMP6 fluorescence in INS-1E cells (Fig. 7A–I; S8A,B), similarly to FASIS at 25 mM glucose (Fig. 7E) and GSIS at 5–9 mM glucose (Fig. 7F). The resulting PA-induced [Ca^2+^]_c_-oscillations at 3 mM glucose also ceased with 10 μM r-BEL (Fig. 7B–J), 2.5 μM etomoxir (Fig. 7C–L), 10 nM SkQ1 (Fig. 7D–M), 2 μM GW1000 (Fig. 7H–N; for reversibility see the subsequent response to 11 mM glucose), and catalase overexpression (Fig. 7G; recovered by KCl). FASIS with 15 μM PA exhibited the predominant high-range oscillation amplitudes (higher time-accumulated [Ca^2+^]_c_) (Fig. 7J–N, left panels; all data in Fig. 7K), resembling the pattern of GSIS at 9 mM glucose [63] (Fig. 7K, right), in contrast to very-low-range oscillation amplitudes at resting 3 mM glucose (Fig. 7J,K,M,O, *gray* in right panels) and nearly no oscillations with nimodipine and overexpressed catalase (Fig. S8B). In fewer cells, [Ca^2+^]_c_-oscillations were also induced with 1 μM Agonist II (Fig. 7I–O).

### 3.7 Insulin secretion upon fatty acid and intralipid administration to wt and iPLA_2_γKO mice

**Fig. 8:**
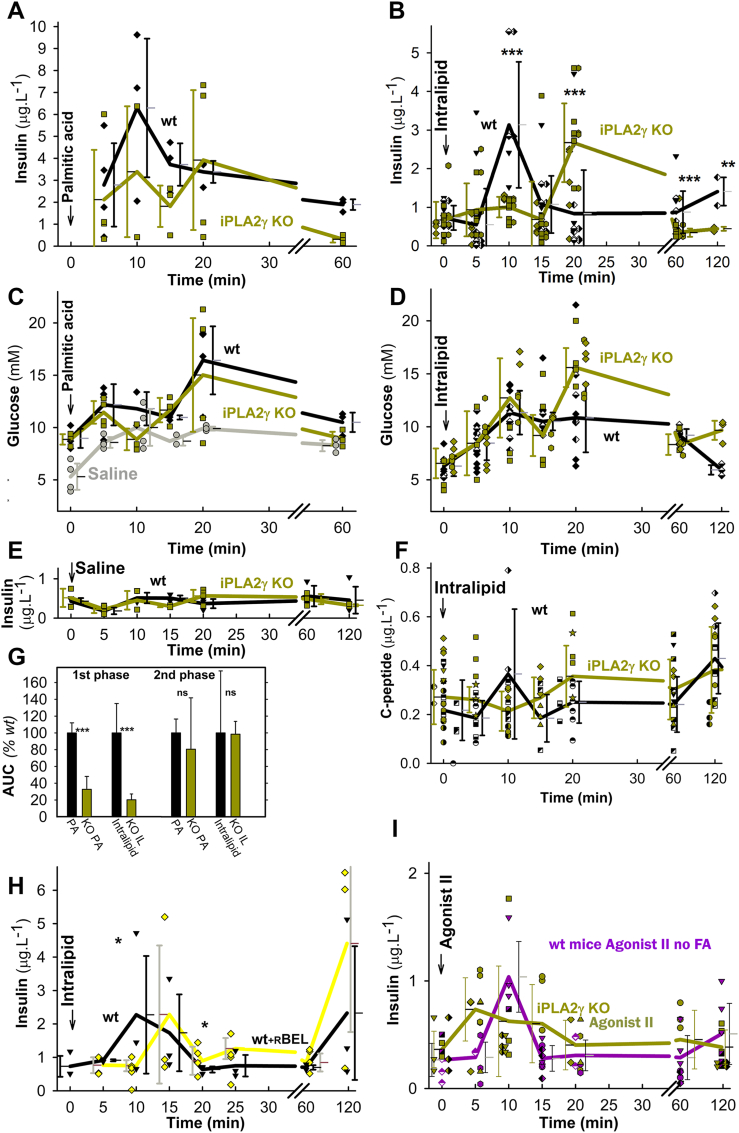
Insulin and c-peptide in blood and glycemia after i.p. administration of palmitic acid and Intralipid *Backcrossed wt mice*: *black*; *iPLA*_*2*_*γKO mice*: *green*. Palmitic acid (100 mg kg^−1^; *left* panels **A**,**C**,**G**; *N* = 10, *n* = 20 for wt or KO; *n* = 20 for C), Intralipid (7.5 μl g^−1^; *right* panels **B**,**D**,**F**,**G**; *N* = 40, *n* = 98 for wt or *N* = 40, *n* = 81 for KO in **B**,**D**; or saline only (**E**; *gray* trace in **C**) were i.p. injected at time zero. AUCs normalized to wt are also shown (**G**), while SDs were derived from AUCs of given time point values + SDs or –SDs. ANOVA: *P < 0.05; **P < 0.01; ***P < 0.001. Each mouse was sampled for blood insulin and glycemia two to three times and time courses were constructed from different times of different mice. The groups of mice investigated are indicated with different symbols. **H**) *wt mice pretreated with* 1 mg kg^−1^r-BEL (*yellow*; *N* = 15, *n* = 30) are compared to *wt* controls (*black*; *N* = 42, *n* = 84). **I) Insulin in blood after i.v. administration of Agonist II in mice**– *Backcrossed wt mice*: *violet, N* = *18, n* = *37*; *iPLA*_*2*_*γKO mice*: *green, N* = *19, n* = *41*. After Agonist II (10 μmol kg^−1^) i.v. administration, each mouse was sampled two to three times for blood insulin and glycemia (glycemia, AUCs, see Supplementary Information Figs. S8D–G) and a time course was constructed from different times of different mice. Groups of mice investigated are indicated by different symbols.

**Fig. 9:**
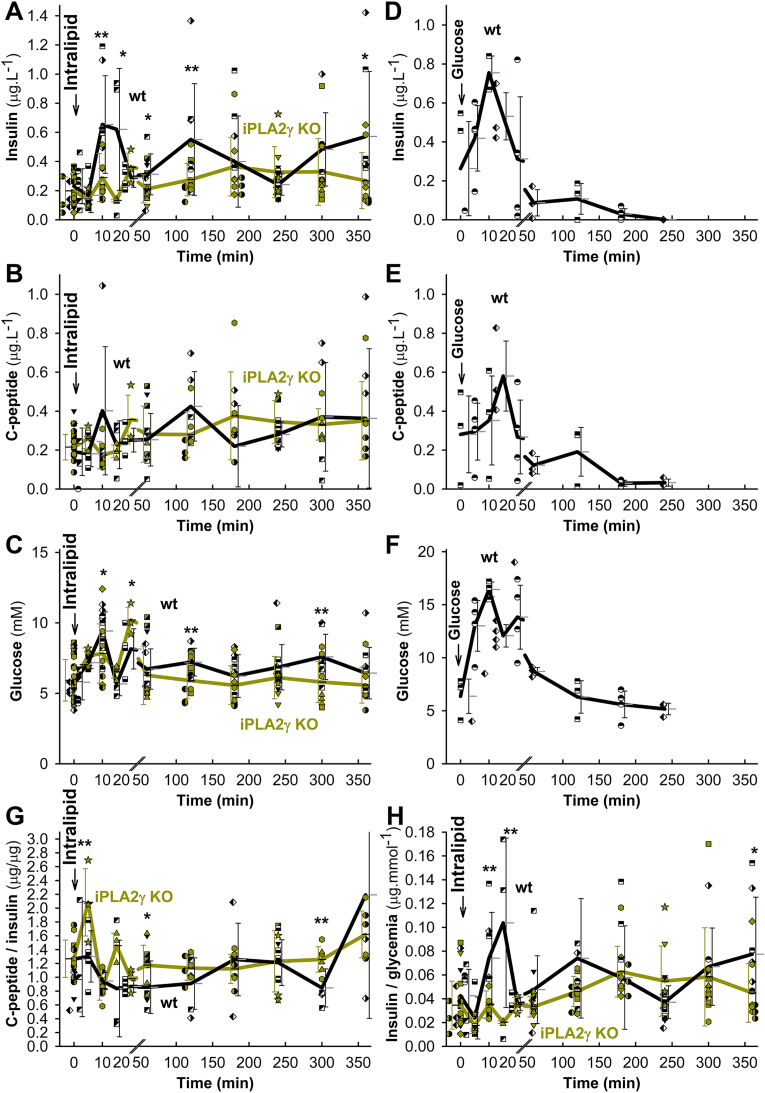
FASIS at extended period *in vivo* compared to GSIS *Backcrossed wt mice*: *black*; *iPLA*_*2*_*γKO mice*: *green*. **A–C**,**G**,**H)** Oral administration of Intralipid (10 μl g^−1^; *n* = 54 for wt or KO) was performed at time zero. Each mice was sampled for blood insulin, c-peptide, and glycemia three times and time courses were constructed from different times of different mice. **D–F)** i.p. administration of glucose (1 mg/g body weight; ∼111 μmol glucose per mouse. The groups of mice investigated are indicated with different symbols. ANOVA: *P < 0.05; **P < 0.01; ***P < 0.001.

GSIS was unaffected in iPLA_2_γKO relatively to wt mice (Figs. S9A–D). The knockouts exhibited neither significantly impaired glucose tolerance nor peripheral insulin resistance (Fig. S9E). The intraperitoneal (i.p.) administration of PA after 6 h of fasting (Fig. 8A) and liposomal triglycerides (Intralipid) after 14 h of fasting (Fig. 8B) induced profound insulin secretion, monitored from the blood in the eye plexus. For Intralipid, besides GPR40, the MAG activation of GPR119 is superimposed. The wt-FASIS 1st phase at 10 min was suppressed by ∼80 % in iPLA_2_γKO mice, in which at ∼20 min the 2nd insulin peak (“delayed 1st phase”) was clearly distinguished, unlike in wt-mice (Fig. 8A and B; for saline controls see Fig. 8E, and for in-phase c-peptide release see Fig. 8F). Early biphasic time course was also reflected for glycemia in both wt and KO mice (Fig. 8C and D; Fig. 9C) and with Agonist II (Figs. S10E–G). Taking insulinemia AUC^2nd^ at 20–60 min, there was no suppression of the 2nd phase in iPLA_2_γKO mice (Fig. 8G). The 1st phase comprised ∼33 % of total released insulin in wt-mice (∼20 % for Intralipid) and ∼7 % in iPLA_2_γKO mice (∼4 % for Intralipid) during 120 min.

Prolonging observations until 6 h, oral Intralipid administration led to triphasic insulin secretion and synchronous c-peptide release also in wt mice with peaks at 10, 120, and ∼360 min (Fig. 9A and B). Formally they were suppressed in iPLA_2_γKO mice. However, one could recognize here rather a delay of the wt peak from 10 min to ∼20 min in iPLA_2_γKO mice, similar shift of the 120 min wt peak to ∼180 min and missing peak at ∼360 min in iPLA_2_γKO mice (Fig. 9A and B; S10A). Since glycemia returned nearly to the initial values (differences wt *vs*. KO were statistically non-significant after 60 min except for peaks at 120 and 300 min; Fig. 9C), we can ascribe the later insulin secretion mainly to a pure FASIS with a minor GSIS contribution. This is supported by the c-peptide-to-insulin and insulin-to-glycemia ratios (Fig. 9G and H; S10B–D). Sole GSIS returns to initial values at ∼180 min (Fig. 9D–F). A c-peptide-to-insulin ratio >0.538 indicates faster insulin internalization relative to c-peptide degradation. Insulin-to-glycemia ratio is higher when insulin secretion occurs at low glycemia. Nevertheless, a minor GSIS contribution could exist around up to 7 mM glycemia peaks at ∼120 min and ∼300 min (Fig. 9C). We thus demonstrate that a modest insulin secretion exists *in vivo*, which is predominantly dependent on FA (lipid) component.

Comparing this with the experimental GSIS (i.p. administration of glucose) in littermates, a typical sharp peak of insulin, c-peptide, and 17 mM glycemia was observed at 10 min, followed by a minor peak only of insulin and c-peptide at 120 min and secretion approaching to after 180 min (Fig. 9D–F; S10B,C). Glycemia also peaked at 20 min, returning to the initial values after 120 min (Fig. 9F).

The delayed and partly suppressed insulin time course with ablated iPLA_2_γ most likely reflects the missing redox-activation of iPLA_2_γ, which in wt-mice cleaves FAs from mitochondrial membranes during FASIS. Indeed, r-BEL administration before Intralipid resulted in a similar effect to iPLA_2_γ ablation, *i.e*. with no ultimate 1st phase, but with a “delayed 1st phase” (Fig. 8H). Missing mitochondrial FAs could be responsible for the delay. Also, Agonist II i.p. administration induced an insulin release, the 1st phase of which was slightly widened (Fig. 8I) or delayed (Figs. S10D–G), but not diminished in iPLA_2_γKO mice.

A concomitant 10 mM glycemia double-peak developed after i.p. and oral Intralipid (12 mM glycemia after PA), as well as Agonist-II i.v. administration, to both wt and iPLA_2_γKO mice (Fig. 8C and D; Fig. 9C), the first 15 min being slightly higher than responses to control i.p. injected saline (Fig. 8E). The saline response originates most likely from central nervous system and/or stress responses. At the same time, the additional glycemia stems from glucose recruitment from the liver or other organs/tissues. This makes it impossible to simulate a net FASIS *in vivo* at glucose below 10 mM unless long timing is surveyed.

## 4 Discussion

**Fig. 10:**
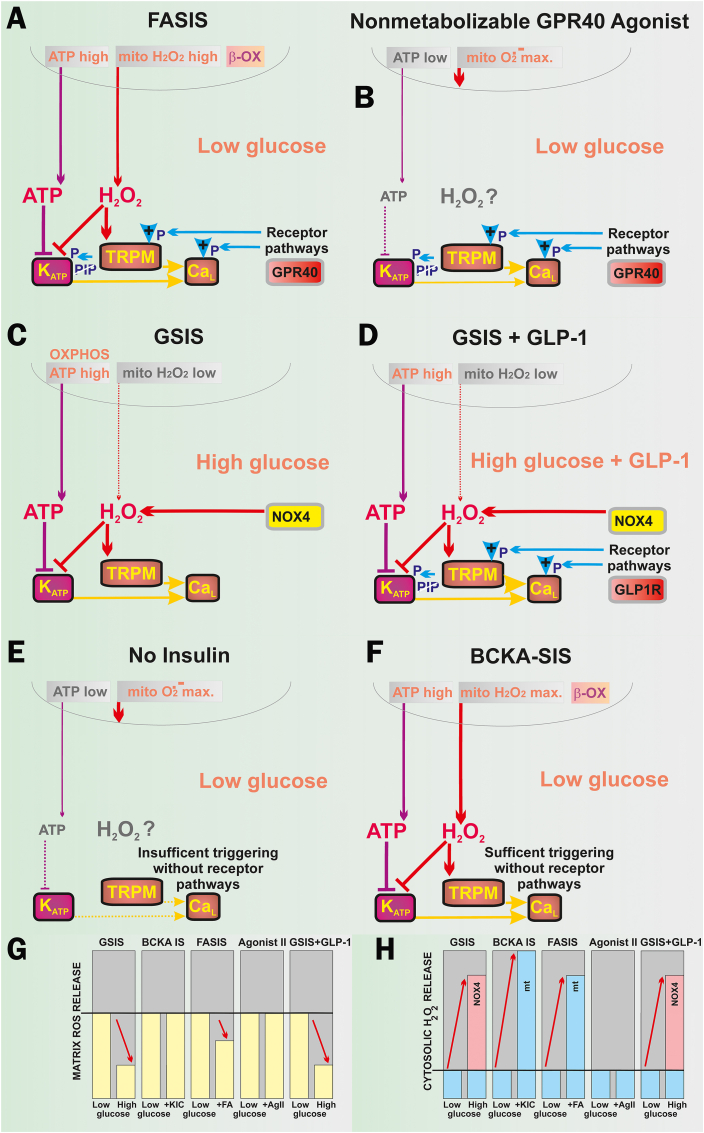
ATP and H_2_O_2_ requirements for insulin secretion by various secretagogues and/or conditions. **A) FASIS**: both mitochondrial ATP and H_2_O_2_ originate from FA β-oxidation (“β-OX”), being required to close K_ATP_, similarly as reported for GSIS [36]. Amplification is ensured by metabotropic GPR40 receptors, activating the canonical G_αq/11_ pathway, involving the Ca^2+^-dependent PLC-hydrolysis of PIP_2_ (“PIP”) [[24], [25], [26], [27], [28], [29], [30], [31], [32], [33]], releasing PIP_2_ from its binding site on K_ATP_ and abolishing its permanent opening [47]. Hydrolyzed DAG and IP3 provide branching into PKC and ER-(IP3R)-involved pathways, while PKC phosphorylates TRPM4 and TRPM5 channels [45], enabling them to participate in the triggering of the Ca_V_-opening cycles. Biased GPR40 activation can initiate the G_αs_-PKA (or EPAC2) pathways, phosphorylating TRPM2 [[39], [40], [41], [42], [43], [44], [45]], K_ATP_, and Ca_V_ (or Munc13-1 and ryanodine receptor [39] on ER). **B) Non-metabolizable GPR40 agonists** at low glucose act at low „resting” ATP levels, but a high superoxide matrix release that is not transferred into the cytosolic H_2_O_2_. The GPR40 canonical/biased pathways ensure a certain level of K_ATP_-Ca_V_-dependent triggering upon the released PIP_2_ (instantly phosphorylated TRPM, Ca_L_, K_V_ channels), providing modest insulin secretion. **C) GSIS** relies on the NOX4-mediated redox signaling (elevated cytosolic H_2_O_2_) plus ATP from OXPHOS (higher ATP/ADP), both essentially required for the K_ATP_-Ca_V_-dependent triggering of IGV exocytosis [[16], [17], [18], [19],^36^,[39], [40], [41], [42]]. Cytosolic H_2_O_2_ is supplied by NOX4 fed by NADPH from the pentose phosphate shuttle and ongoing redox shuttles [42,60]. Due to the latter, mitochondrial H_2_O_2_ release to the matrix decreases dramatically [37]. **D) GSIS amplified by incretins**, such as GLP-1, proceeds again at NOX4-mediated H_2_O_2_ plus high ATP. However, it now involves a canonical amplification by G_αs_-PKA and EPAC2 pathways with the consequences described in A). **E) Low-glucose conditions with no other metabolites,** however, do not create enough of ATP, or NOX4-mediated H_2_O_2_. A high superoxide matrix release is not transferred into a sufficient cytosolic H_2_O_2_. Moreover, there is also no receptoric amplification. Due to all these three reasons, insulin is not secreted. **F) Low-glucose conditions with 2-keto-isocaproate (KIC):** or 2-ketoisovalerate or 2-ketomethylvalerate, *i.e*. with branched-chain ketoacids (BCKAs) as secretagogues, produce BCKA-stimulated insulin secretion, involve the low-glucose conditions plus β-like oxidation (“β-OX”) which produce both high OXPHOS (sufficient ATP and/or ATP/ADP) plus high H_2_O_2_ release into the cytosol [36]. **G,H)** Tentative schemes of matrix (**G**) and cytosolic redox (**H**) changes [36,37,60] in an arbitrary scale, emphasizing changes.

The acute redox signals in cells originating from mitochondria were considered to be unlikely [68] or being exceptional under specific metabolic conditions [69] or created artificially [70]. Now we demonstrated the existence of two types of redox signaling, essential for FASIS at low glucose: *i*) *redox signaling from mitochondria to the plasma membrane*, ensuring K_ATP_ closure, thus triggering the metabolic FASIS; and *ii*) *intramitochondrial redox signaling*, activating phospholipase iPLA_2_γ/PNPLA8. Besides the existence of FASIS *in vivo*, we also demonstrated GPR40-receptor stimulation *in vivo* by iPLA_2_γ-cleaved mitochondrial FAs [37]. Observing H_2_O_2_ penetration into the extracellular space with Amplex UltraRed upon FASIS and the insulin release stimulated with only H_2_O_2_, we hypothesize that redox signaling is an essential component of the insulin secretion mechanism (Fig. 10). Elevated ATP (ATP/ADP) alone is insufficient for insulin secretion, e.g. with mitochondria-targeted antioxidant SkQ1, or carnitine palmitoyltransferase-1 inhibitor etomoxir, both of which block FASIS. Both allowed relatively high OXPHOS and ATP, but no insulin secretion.

The H_2_O_2_-activated phospholipase iPLA_2_γ/PNPLA8 cleaves both saturated and unsaturated FAs from mitochondrial phospholipids [48]. A possible iPLA_2_γ/PNPLA8 partial population residing in the endoplasmic reticulum would also be compatible with our data. The released free FAs diffuse up to the plasma membrane, where they mainly activate GPR40-receptors [37], while also being partially consumed by β-oxidation, aiding the metabolic triggering of insulin secretion. With ablated iPLA_2_γ/PNPLA8, there is neither endogenous GPR40 activation nor a surplus β-oxidation supply to OXPHOS. The residual FASIS in iPLA_2_γKO-PIs is given by the metabolic (fuel) component, predominantly during the 1st phase and entirely during 2nd phase, respectively, as judged from a partial and slight GW1100 inhibition, respectively. Both metabolic (fuel) and GPR40-receptoric (non-fuel) FASIS are concomitant to the Ca_L_-channel-mediated Ca^2+^-influx into the cell cytosol.

For FASIS, the latter depends on the redox signaling from mitochondria ensuring a cooperative K_ATP_ closing, together with ATP, with H_2_O_2_ formed by the concomitant OXPHOS, supplied by FA β-oxidation [36] (Fig. 10A). Elevated OXPHOS is not strictly required for non-metabolizable GPR40-agonists (Fig. 10B), but still their down-stream pathways allow the K_ATP_-dependent triggering (Fig. 4C; Fig. 7I–O). Probably downstream GPR40 pathways induce the K_ATP_-closure, because of the Gαq_/11_- and Ca^2+^-dependent PLC-hydrolysis of PIP_2_ [[24], [25], [26], [27], [28], [29], [30], [31], [32], [33]] that leads to the PIP_2_ release from K_ATP_, which abolishes its permanent opening [47]. In any case, the facilitation of K_ATP_-closing and TRPM/NSCC activation (both required for Ca_L_ opening [[39], [40], [41], [42], [43], [44], [45]]), which is ensured by pathways downstream of GPR40, requires further investigation. Finally, since the GPR40 pathway is not stimulated in the absence of its agonists at low glucose, there is no insulin exocytosis (Fig. 10E).

Acute GPR40 activation proceeds *via* the Gαq_/11_/IP3 or Gαq_/11_/DAG pathways [22,[25], [26], [27], [28], [29], [30], [31], [32], [33]] with biased Gαs/cAMP/PKA or Gαs/cAMP/EPAC2 pathways [44]. Upon FASIS, redox-activated NSCCs, in conjunction with their PKA-mediated (TRPM2) [[39], [40], [41], [42], [43], [44], [45]] and PKC-mediated phosphorylation (TRPM4,5; TRPC3) may contribute to triggering Ca_V_-dependent [Ca^2+^]_c_-oscillations at lower ATP levels, generated at low glucose. We conclude that either *i*) a redox signal (elevated cytosolic ROS release) plus elevated ATP (ATP/ADP) are required for insulin secretion in general (Supplementary Table 3); or *ii*) PIP_2_ release from K_ATP_ plus proper phosphorylation of channels (K_ATP_, TRPM, Ca_L_) should occur [[39], [40], [41],[43], [44], [45]].

The ATP rise upon FASIS is about equal to that upon GSIS in INS-1E cells, but mitochondrial superoxide matrix release is lower than at low glucose, due to UCP2-iPLA_2_γ antioxidant synergy [37,48,59]. However, the latter does not exist with silenced [37] or ablated iPLA_2_γ. Therefore, iPLA_2_γ-KO PIs exhibit similar extracellular H_2_O_2_ release to wt-PIs upon FASIS. The redox signal mechanisms involved upon FASIS, including the targets of the redox signaling (K_ATP_, TRPMs), are likely to be equal to those acting upon GSIS [36,39,40], except for the source, i.e. NOX4. GSIS relies on both, the NOX4-mediated redox signaling (elevated cytosolic H_2_O_2_) plus on ATP from OXPHOS (Fig. 10C); both of which are essentially required for the K_ATP_-Ca_V_-dependent triggering of IGV exocytosis [[16], [17], [18], [19],^36^,[39], [40], [41], [42]]. A similar metabolic (fuel) FASIS component, but with redox signals initiated by mitochondrial β-oxidation, remained predominant in iPLA_2_γKO mice and during the 2nd phase in wt-mice. Finally, GSIS can be amplified by incretins, such as GLP-1 (Fig. 10D), and then all three components, redox, metabolic, and receptoric participate in its mechanism as for FASIS.

The essential requirement of mitochondrial redox signaling for insulin secretion is suggested by FASIS prevention by mitochondrial antioxidant SkQ1, Trolox, or catalase overexpression. Lower „resting“ ATP levels, generated by 5.5 mM glucose in PIs, were insufficient to trigger insulin without FAs (Fig. 10E). Mitochondrial H_2_O_2_ redox signaling is also involved in 2-oxo-isocaproate-stimulated insulin secretion [36] (Fig. 10F). In this case, it proceeds at high ATP and without any receptoric component, since the β-like-oxidation of branched-chain keto-acids (BCKA), such as 2-oxo-isocaproate (*aka* 2-keto), produces both [36] ATP and H_2_O_2_. The homeostasis of redox buffers and the redox relay system of peroxiredoxin in pancreatic islets [2,17,39,40] is set so that H_2_O_2_, generated from the elevated superoxide upon FA β-oxidation, reaches the plasma membrane, as documented by Amplex-UltraRed perifusion. This is similar for BCKA β-like-oxidation [36].

Fig. 10G,H summarizes ROS/H_2_O_2_ releases with various secretagogues, as measured here or published elsewhere [36,37,60], for changes in the mitochondrial matrix as well as the β-cell cytosol. The common source for both redox signals upon FA β-oxidation stems from mitochondrial superoxide formation, which is faster than the steadily decreasing superoxide upon GSIS conditions [60] (Fig. 10H). This superoxide source is oriented and enhanced so that after MnSOD-mediated dismutation the resulting H_2_O_2_ can reach the plasma membrane. The source of this superoxide is probably at the Complex I site I_F_. In INS-1E cells, maximum superoxide release to the mitochondrial matrix occurs at low glucose, whereas it is reduced to ∼40 % upon GSIS due to the operation of redox shuttles [60]. At low glucose, the lower H_2_O_2_ penetrating to the cytosol, together with “resting” ATP, does not provide a sufficient milieu for triggering [Ca^2+^]_c_-oscillations also due to the lack of the receptoric component unless the GLP-1 action is superimposed (M. Jabůrek, unpublished).

The experimental FASIS and c-peptide release was initiated in PIs and mice at low fasting glucose; and in mice persisted after a few minutes at ∼10 mM glucose, continued at ∼150 min when glucose returned to low concentrations, but lasted over 360 min. The initial extra glucose was released into the blood from peripheral tissues/organs as a response to suddenly increased FAs or even Agonist II. Consequently, *in vivo* “experimental FASIS” involves secreted insulin due to the FA-mediated signaling through metabotropic receptors (GPR40, GPR120 for FAs with C > 6; GPR41, GPR43 for FAs with C < 6) [24,30] plus from the concomitant combined metabolic (OXPHOS) component, supplied by FA β-oxidation, but accompanied by GSIS, which is given by the intermediate ∼10 mM glucose. The claimed exclusive dependence on a primary glucose rise [[23], [24], [25], [26], [27], [28], [29], [30], [31], [32], [33]] turned out to be unnecessary [34]. We show the opposite sequence of events: at first a rapid primary FA-stimulation of FASIS with slightly delayed glucose recruitment into the bloodstream, inducing concomitant GSIS, superimposed onto the initial FASIS. Nevertheless, we clearly demonstrated the existence of FASIS *in vivo* at low glucose at 150–360 min, when oral Intralipid was administered to mice (Fig. 9A). The physiological meaning of this lies in allowing glucose penetration into cells of peripheral tissues also at lower glucose at later postprandial times, specifically with fat-containing meals.

Unlike in our supra-physiological experiments, several responses to a typical fat meal can be expected *in vivo* [46]: *i*) net GSIS; *ii*) postprandial responses of pancreatic β-cells to incretins, secreted upon the intestinal digestion of meal [39,40]; and *iii*) the direct insulin-stimulatory role of free FAs and 2-monoacylglycerol for β-cells. Since chylomicrons come to the pancreas at >1 h after the fat meal in rodents (peaking after two to 4 h, depending on the fat amount in humans, see Fig.7.11 in Ref. [46]), the first two responses should be distinguished from the responses to FAs and MAG, which are thought to occur during the late GSIS 2nd-phase, when glycemia approaches to the fasting glycemia. Hypothetically, such physiological FASIS might contribute to the fasting insulin levels [71] and should be further studied *in vivo*.

In conclusion, we have demonstrated the existence of a redox signal that originates from mitochondria and reaches the plasma membrane upon FA β-oxidation. In pancreatic islet β-cells, this signal enables fatty acid-stimulated insulin secretion (FASIS) at a low glucose concentration, which individually does not stimulate insulin secretion. In isolated PIs and in mice, the 1st FASIS phase was largely dependent on mitochondrial redox-activated phospholipase iPLA_2_γ, acting so that it enriches the free FA pool for GPR40-signaling by mitochondrial FAs, which also partly supplies a surplus for β-oxidation. The potentiating GPR40-signaling was largely dependent on the essential “metabolic” FASIS component, ensured by mitochondrial redox (H_2_O_2_) signaling together with elevated ATP, both cooperatively closing ATP-sensitive K^+^ channels. Nevertheless, minor insulin secretion responses were observed with non-metabolizable GPR40-agonists. We conclude that the intra-mitochondrial and mitochondria-to-plasma-membrane redox signaling are two crucial integral FASIS components, and that FASIS exists *in vivo* as a physiological, postprandially delayed, event.

## CRediT authorship contribution statement

**Martin Jabůrek:** Conceptualization, Formal analysis, Funding acquisition, Investigation, Methodology, Resources, Writing – review & editing. **Eduardo Klöppel:** Investigation. **Pavla Průchová:** Investigation, Methodology. **Oleksandra Mozheitova:** Investigation. **Jan Tauber:** Investigation. **Hana Engstová:** Formal analysis, Investigation, Methodology. **Petr Ježek:** Conceptualization, Data curation, Formal analysis, Funding acquisition, Project administration, Supervision, Validation, Visualization, Writing – original draft, Writing – review & editing.

## Declaration of competing interest

There are no competing financial interests.
