# Supplementary material for: Mitochondria to plasma membrane redox signaling is essential for fatty acid β-oxidation-driven insulin secretion
Source: Redox Biol. 2024 Jul 23;75:103283. doi: 10.1016/j.redox.2024.103283 (PMC11332078; doi:10.1016/j.redox.2024.103283)
Supplement: Multimedia component 1 [file mmc1.pdf]

Supplementary material for

Mitochondria to plasma membrane redox signaling is essential for fatty acid β-oxidation-driven insulin secretion

Martin Jabůrek[1], Eduardo Klöppel[1], Pavla Průchová[1], Oleksandra Mozheitova[1],

Jan Tauber[1], Hana Engstová[1] and Petr Ježek[1,*]

[1]Department of Mitochondrial Physiology, No.75, Institute of Physiology of the Czech Academy of Sciences, Prague, 14220, Czech Republic

Corresponding author:** ***jezek@biomed.cas.cz

Table S1 Key Resources

Part I Creation of PNPLA8 knock-out (iPLA2γKO) mice

Part II Meaning of experimental data for islet perifusion for FASIS and GSIS in iPLA2γKO murine PIs

Part IIA Meaning of ^{13}C tracking from U-^{13}C-palmitic acid

Part III Insulin secretion responses to glibenclamide and no FASIS inhibition with S1QEL and S3QEL in murine PIs

Part IV Respiration of pancreatic islets from wt *vs*. iPLA2γ KO mice

Part V Carnitine palmitoyl transferase 1 silencing

Part VI Monitoring of cytosolic redox signal upon GSIS *vs*. FASIS and induction of insulin secretion with pro-oxidants

Part VII Responses of Ca^{2+} oscillations to glucose in wt *vs*. iPLA2γKO murine PIs

Part VIII GSIS and absence of insulin resistance in iPLA2γKO mice

Part IX Details of *in vivo* FASIS in wt *vs*. iPLA2γKO mice

Part X Derived conditions for insulin secretion

Table S1 Key resources table

Chemicals		
**Reagent**	**Source**	**ID**
Acetonitrile	Supelco (2024)	1.00029.2500
Agonist-II (3-(4-((2,6-dichloropyridin-4-yl)ethynyl)phenyl)propanoate)	Merck/Calbiochem (2022)	371721-10MG
Aminooxyacetate	Sigma Aldrich (2010)	C13408
6-aminonicotinamide	Sigma Aldrich (2010)	08820-16
Ammonium formate	Sigma Aldrich (2024)	70221-100G-F
Amplex™ UltraRed	Thermo Fisher Scientific (2020)	A36006
Antimycin A from Streptomyces sp.	Sigma Aldrich (2022)	A8674-25MG
Bio-Gel P4	Bio-Rad (2017)	150-4120
Bovine serum albumin, BSA	Sigma Aldrich (2018)	A3912-100G
Bovine serum albumin FFA free	Sigma Aldrich (2016)	A7030-100G
Bromopalmitic acid (2-Bromohexadecanoic acid)	Merck (2024)	21604-1G
Calcium chloride	Sigma Aldrich (2015)	C3306
CAY10587	Caymann (2022)	13143
Cromakalim	Sigma Aldrich (2015)	C1055-10MG
d4-tyrosine	CDN isotopes (2024)	D-7498
Decanoic acid (capric acid)	Sigma Aldrich (2015)	C-1875 100G
Decyltriphenylphosphonium bromide, Decyl-TPP	Biosynth Carbosynth (2022)	HBA33943
H2DCFDA (2',7'-dichlorodihydro-fluorescein diacetate), DCF	Thermo Fisher Scientific (2016)	C6827
Diazoxide	Sigma Aldrich (2013)	D9035-250MG

EDTA	Sigma Aldrich (2015)	ED-500g
EGTA	Sigma Aldrich (2017)	E4378-100G
Etomoxir	Sigma Aldrich (2022)	236020-5mg
Fetal calf serum, FCS	Seralab (2016)	Gem 100-506 Bovine calf serum
Glucose	Sigma Aldrich (2016)	G8270,D-(+)-glucose-1kg
^{14}C-Glucose	UVVR, Řež, Czech Republic	N/A
Glibenclamide	Sigma Aldrich (2012)	G0639 Glybenclamide
Glutaraldehyde, 50 %	EMS (2017)	16320
L-glutamine	Cambridge isotope laboratories (11/2016)	CLM-3612-1 L-GLUTAMINE (1-13C, 99%)
GW1100	Cayman (2022)	10008908
H_2O_2, stabilized	Sigma Aldrich (2017)	216763-500ML-M, Hydrogen peroxide solution
HEPES	Sigma Aldrich (2017)	H3375
Hexanoic acid sodium salt (caproic acid)	Merck (2024)	C4026
Indol-2,4,5,6,7-d5-3-acetic acid	CDN isotopes	D-2203
Intralipid	Sigma Aldrich (2015)	I-141
Krebs–Ringer bicarbonate	Cambride isotope laboratories (2017)	DLM-3487-PK: 0.5 G CITRIC ACID (2,2,4,4-D4, 98%)
Lauric acid	Sigma Aldrich (2015)	L-4250 100G
Linoleic acid	Sigma Aldrich (2017)	L1376-5G
Lipofectamine 2000	Thermo Fisher Scientific (2016)	11668500
Magnesium chloride	Sigma Aldrich (2016)	M8266
Mercaptoethanol	Sigma Aldrich (2012)	63689-25ml-F
Methanol ≥99.8%, HiPerSolv	VWR (2023)	20864.320

CHROMANORM® for HPLC		
MitoSOX-Red	Thermo Fisher Scientific (2022)	M36008
Nimodipine	Tocris (2012)	0600
Oleic acid	Sigma Aldrich (2016)	O1008-1G
Oligomycin A	Sigma Aldrich (2016)	O4876
Oligofectamine	Thermo Fisher Scientific (2016)	12252-011
2-oxo-isocaproate	Sigma Aldrich (2016)	K0629 Sodium 4-methyl-2-oxovalerate
Oxythiamine chloride hydrochloride	Sigma Aldrich (2015)	O4000
Palmitic acid	Sigma Aldrich (2015)	P0500
Palmitic acid – $^{13}C16$	Sigma Aldrich (2024)	605573
Paraformaldehyde, 32%	Biogen (2017)	15714
Penicilin, streptomycin	Sigma Aldrich (2017)	P0781,100ml
Peroxidase type II from horseradish	Sigma Aldrich (2019)	P8250-25KU
PicoGreen Assay	Thermo Fisher Scientific (2016)	P7581
Poly-L-lysine hydrobromide	Sigma Aldrich (2016)	P9155
Potassium chloride	P-lab (2021)	Q 04101
R-bromoenol-lactone	Cayman (2015)	10006800
Rotenone	Sigma Aldrich (2017)	R8875-1G
RPMI 1640 medium	Institute of Molecular Genetics, Prague	N/A
S1QEL	Life Chemicals	(shipped from Spoluka Chemical Co., Kiev, Ukraine)
S3QEL	Life Chemicals	(shipped from Spoluka Chemical Co., Kiev, Ukraine)
S-bromoenol-lactone	Cayman (2012)	10006801

SkQ1 (10-(6'-plastoquinonyl)decyltriphenylphosphonium)	Mitotech LLC, Moscow, Russian Federation	N/A
Sodium azide	Sigma Aldrich	71289
Sodium chloride	Lachner (2019)	30093-APO
Sodium phosphate monobasic	Sigma Aldrich (2016)	S5011
Sodium phosphate dibasic	Sigma Aldrich (2016)	S0876
Sodium Pyruvate	Sigma Aldrich (2016)	P2256-5G
Sodium stearate	Sigma Aldrich (2016)	S3381
Stigmatellin	Fluka/ Sigma Aldrich (2012)	85865
tert-Butylhydroperoxide	Sigma Aldrich (2018)	458139
Trizma base	Sigma Aldrich (2016)	T6066
Trolox (6-hydroxy-2,5,7,8-tetramethylchroman-2-carboxylic acid)	Cayman	10011659
Val-Tyr-Val	Merck	V8376
X-treme Gene DNA reagent	Roche	06365779001
**Experimental models: cell lines and animal strains**		
Rat INS-1E cells	AddexBio , San Diego, CA	C0018009
Mouse: iPLA$_2\gamma$ knockout „iPLA$_2\gamma$KO“	generated using transcription activator-like effector nucleases (TALENs) starting with C57Bl6/N mice	This work
Mouse: wild-type backcrossed >10 generations into the PNPLA8-knockout mice background „wt“		This work

Mouse: NOX4 knockout „NOX4 KO“	See Ref. 36	RRID: Mouse Genome Informatics [MGI]:4838554
Mouse: wild-type backcrossed >10 generations into the NOX4 knockout mice background „wt“	See Ref. 36	
**Antibodies**		
Glucagon antibody	Abcam (2018)	RRID:AB_297642, ab10988
Insulin antibody	Cell Signaling Technology (2018)	RRID:AB_10694498, 4590s
**Plasmids, siRNAs**		
pZeoSV2(+) with human catalase (pZeoSV(+)-h catalase)	A kind gift from C. Glorieux and Prof. J.B. Verrax, Universite´ Catholique de Louvain, Belgium	
pCS2+HyPer7, mammalian expression vector allowing HyPer-7 expression in cytosol under the control of CMV promoter	Addgene	136466
pGP-CMV-GCaMP6s, vectors expressing the GCaMP6 fluorescent Ca^{2+} indicator	Addgene	40755
Silencer Select siRNA	ThermoFisher	s130674 Cpt1a
**Kits**		
ATP Bioluminescence Assay kit HSII	Sigma Aldrich (2020)	11699695001
Mouse C-peptide ELISA kit	Crystal Chem (2023)	90050
Glucometer instrument and stripts	Roche (2016)	Accu-Chek Performa Nano
Insulin Mouse-Insulin-High-Sensitivity-ELISA kit (BioVendor, Brno, Czech Republic)	Biovendor	RAI005R
Insulin Rat-Insulin-High-Sensitivity-ELISA kit (BioVendor, Brno, Czech Republic)	Biovendor	RAI006R
Insulin ultrasensitiv mouse ELISA kit (Mercodia, Uppsala, Sweden)	Mercodia	10-1249-01
Seahorse XFe24 Islet Capture FlucPak	Agilent	103518-100

Tl^+ flux kit	Thermo Fisher Scientific	FluxOR potassium ion channel assay
**Column**		
XBridge Premier BEH C18 Van Guard FIT (150 x 2.1 mm, 2.5 µm)	Waters	186009845
**Software**		
CorelDRAW 2017	Corel Corporation, Ottawa, Canada	N/A
Image J	NIH Image	Open source
Ellipse	ViDiTo, Košice, Slovakia	N/A
SigmaPlot 9.0.	Systat Software, San Jose, CA	N/A
SigmaStat 3.1.	Systat Software, San Jose, CA	N/A

Part I I Creation of PNPLA8 knock-out (iPLA2γKO) mice

iPLA2γ/PNPLA8 knockout (KO) mice were generated from C57Bl6/N mice, using transcription activator-like effector nucleases (TALENs) [65], as previously described [64]. TALENs were designed to target the *Pnpla8* exon-3 to eliminate the *Xba*I restriction site (Fig. S1). DNA from tails of young mice was analyzed by PCR restriction-fragment-length polymorphism. Purified PCR products were digested with *Xba*I (ThermoFisher, Waltham, MA, USA). To select appropriate mice for breeding, the PCR products were subcloned into the pGEM®-T Easy Vector System (Promega, Madison, WI, USA). Sequencing only selected those mice bearing a 13 base-pair long deletion in exon-3 for further breeding and was performed using M13 reverse and forward sequencing primers. This deletion produces a premature stop codon in the fourth exon, making it unable to express *Pnpla8*. The wild-type (wt) mice used were those backcrossed >10 generations into the PNPLA8-knockout mice background.

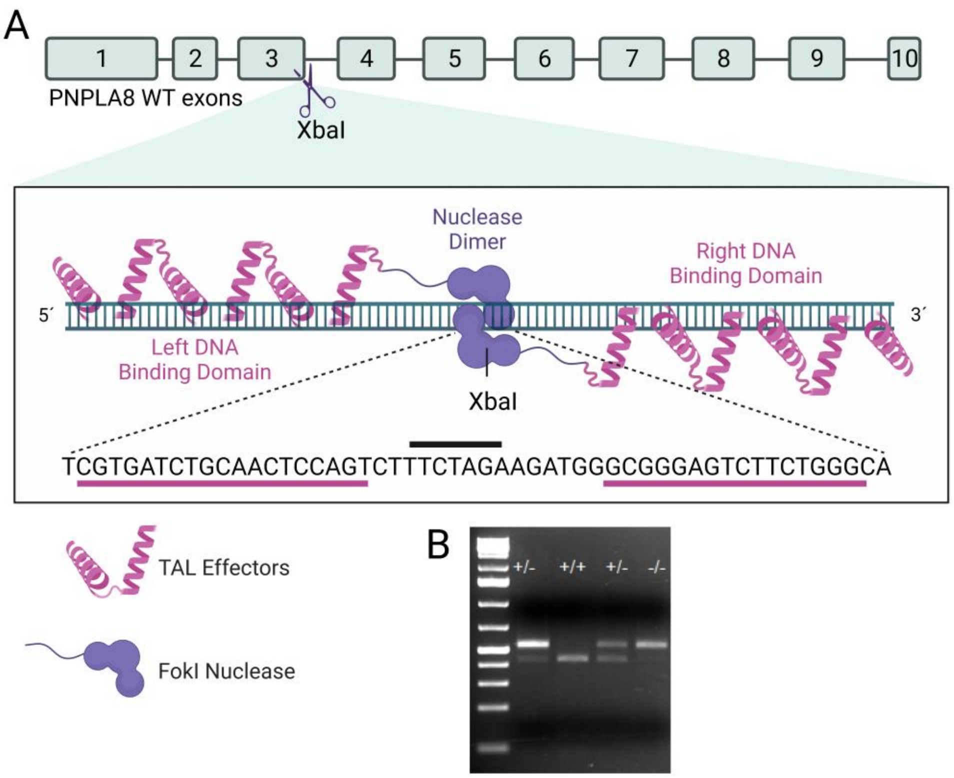

Figure S1 **(A) Construction of PNPLA8 knock-out mice by targeting the PNPLA8 gene, exon 3**, using TALEN (FokI nuclease). Its cleavage deletes 13 base pairs, including the *Xba*I restriction site and creates a premature stop codon in exon 4. **(B) Verification** by PCR restriction-fragment-length polymorphism of the genomic DNA purified from mouse tails; +/+ wild type, +/- heterozygote, -/- knock-out. Primers PNPLA8 forward were AAGAGTCCGCCCGAAGAACAG and PNPLA8 reverse AGGCTTTGCATTCCCCACTTT. *Xba*I restriction provides a wt band of ~400, whereas the KO band is ~500.

Part II Meaning of experimental data for islet perifusion for FASIS and GSIS in iPLA2γKO murine PIs

Islet perifusion data are expressed in the same way as the direct output of the experiment. Formally and for simplicity, the y-axis units of the perifusion time courses are designated as „ng/μgDNA”, referring to a yield of insulin, quantified as ng of insulin *per* number of islet cells, which contain 1 μg of DNA. However, since we actually quantified the amount of insulin secreted during the preceding 2 min for each time point, these data, representing an instant yield, can be recalculated into insulin release rates. The correct dimension of the y-axis units in the experimental plot displaying the instant yield is ng of insulin accumulated within the 2-min interval of perifusion sampling, normalized to the number of islet cells, which contains 1 μg DNA (*i.e*. units of ng *per* 2 min·μgDNA). Moreover, time intervals were set to 5 min after 25 min, hence the correct dimension of the y-axis units is ng of insulin accumulated within the 5-min interval of perifusion sampling, normalized to the number of islet cells, which contains 1 μg DNA (*i.e*. units of ng/5 min·μgDNA). When integrated over a chosen time-period (equal to the period of the 1st phase or arbitrarily selected for the 2nd phase), the resulting AUCs are expressed in units, which are truly ng/μgDNA. Fig. S2 shows how the plots for FASIS (Fig. S2A, recalculated from Fig. 1A) are re-plotted as insulin secretion rates at the given moment.

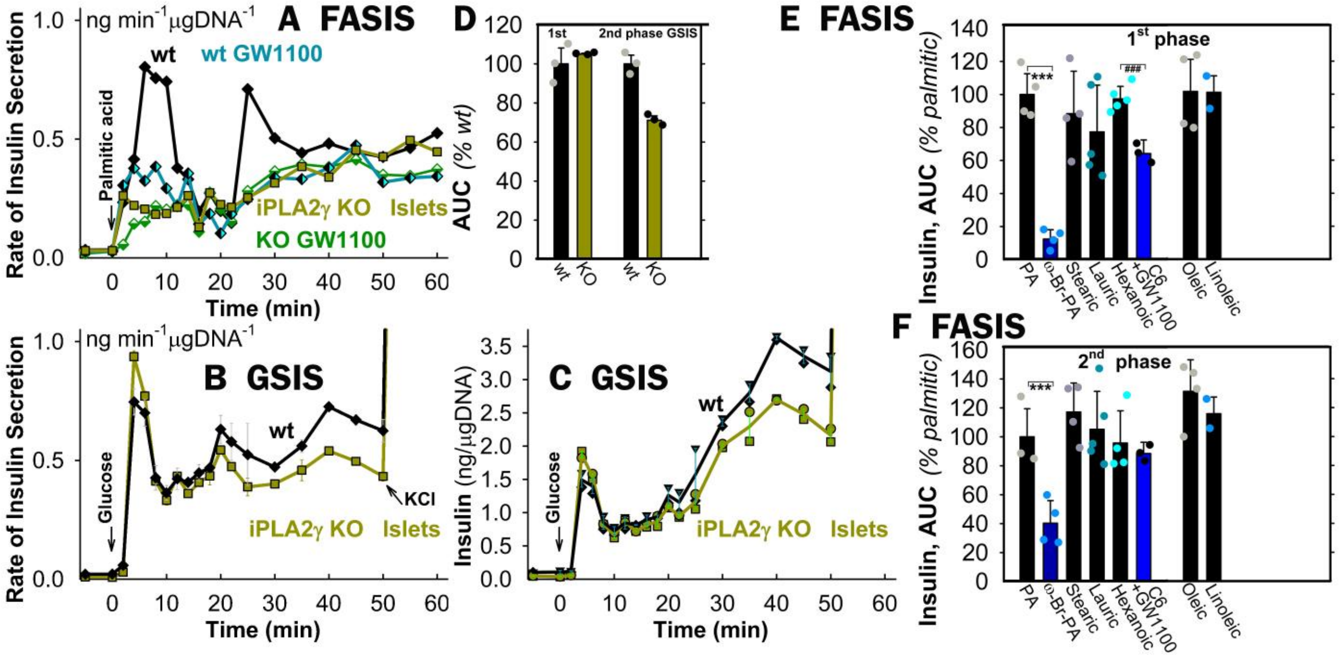

Figure S2 Perifusion data recalculated as insulin secretion rate at the given moment, comparison of various FAs – Raw experimental data of Fig. 1A for FASIS and panel **C** for GSIS were recalculated to obtain the instant rate of insulin secretion (**A**,**B**), calculated as the average rate over the preceding 2 min (preceding 5 min after 25 min of the run). PIs of backcrossed wt mice (*black, or semi-filled symbols*) and iPLA2γKO mice (*green*) were perifused as described in the Fig. 1A legend. When indicated, 1 μM GW1100 (*black/cyan* or *dark green/white semi-filled* symbols) was present. **D**) Comparison of AUCs for FASIS and GSIS. (**E**,**F**) Total insulin amounts secreted at the 1st phase (**E**) and 2nd phase (**F**) upon PI perifusion with FAs giving equal H_2O_2 response in 30 μM BSA, *i.e*. 50 μM PA, wBr-PA, linoleic; 65 μM stearic; 70 μM oleic; 90 μM lauric; and 180 μM hexanoic acid.

Next, data for glucose-stimulated insulin secretion (GSIS) are present for murine PIs from wt and iPLA2γKO mice (Fig. S2B, recalculated from original data displayed in Fig. S2C). These data demonstrate that the peak rate for wt PIs is similar for FASIS and GSIS. Fig. S2D then shows the calculated AUCs.

Also, we compare AUCs for selected FAs (Fig. S2E,F) at the doses theoretically giving similar free FA concentrations. To calculate them (Table S2), we used the following formula:

$$\mathbf{[FA]_{unbound}} = (\, K_d \cdot \mathbf{[FA]_{total}}\,) \;/\; (\, N_{binding\ sites} \cdot \mathbf{[BSA]_{total}} - \mathbf{[FA]_{total}}\,) \qquad \{eq.1\}$$

from the Reference [72] Huber *et al.* (2006) Biochemistry 45, 14263–14247.

**Fatty acid**	K_d (nM)	$N_{binding\ sites}$	$[FA]_{total}$	$[FA]_{unbound}$ (nM)
Palmitic acid	8	6.9	50	2.55
Stearic acid	4.1	5.8	65	2.44
Oleic acid	5.9	7.4	70	2.72
Linoleic acid	13	7.3	50	3.85

Table S2 Calculated free concentrations for selected FAs and their doses

Part IIA Meaning of ^{13}C tracking from U-^{13}C-palmitic acid

To track downstream metabolites of fatty acid β-oxidation, MS of CoA derivatives is nearly impossible. Hence, carnitines, e.g. those converted from CoA by carnitine O-acetyltransferase (CRAT) are tracked instead. Besides the 1.1% of natural content of ^{13}C, butyryl-carnitine with four ^{13}C atoms (M+4) can arise exclusively due to β-oxidation from the added U-^{13}C-palmitic acid (i.e. palmitic acid with all C atoms being ^{13}C). This is shown on the scheme S1 below (^{13}C atoms are depicted in blue). Similarly, CRAT can metabolize acetyl-CoA formed in each cycle of β-oxidation to acetyl-carnitine (C2-carnitine) and hence any acetyl-carnitine with two ^{13}C atoms (M+2) should arise exclusively from β-oxidation of U-^{13}C-palmitic acid.

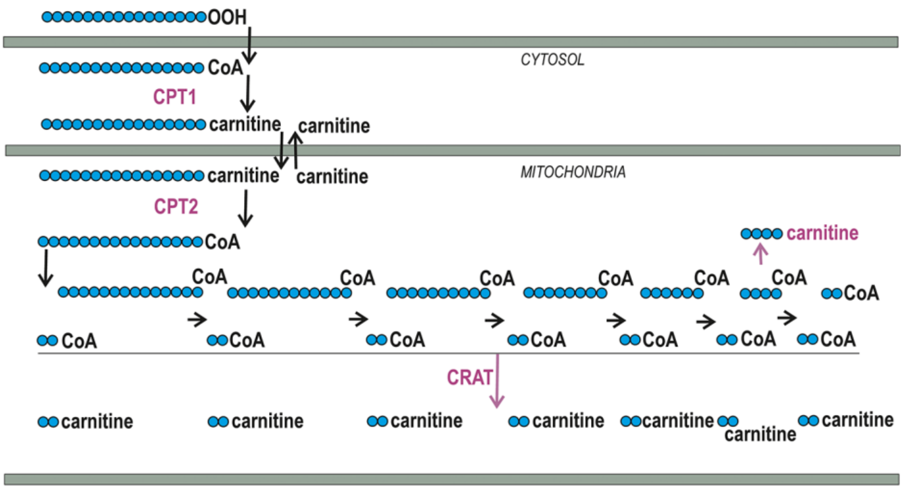

Scheme S1 Metabolite tracking upon β-oxidation of U-^{13}C-palmitic acid

Part III Insulin secretion responses to glibenclamide and no FASIS inhibition with S1QEL and S3QEL in murine PIs

Glucose-stimulated insulin secretion (GSIS) assayed during PI perifusion (Fig. S2) had a virtually equal 1st phase in wt and iPLA2γKO PIs, demonstrating intact GSIS in iPLA2γKO PIs. The 2nd-phase yield was somewhat lower in iPLA2γKO PIs. Analogously, the observed unchanged responses to glibenclamide reflect the intact function of K_{ATP}-channels in iPLA2γKO PIs (Fig. S3A).

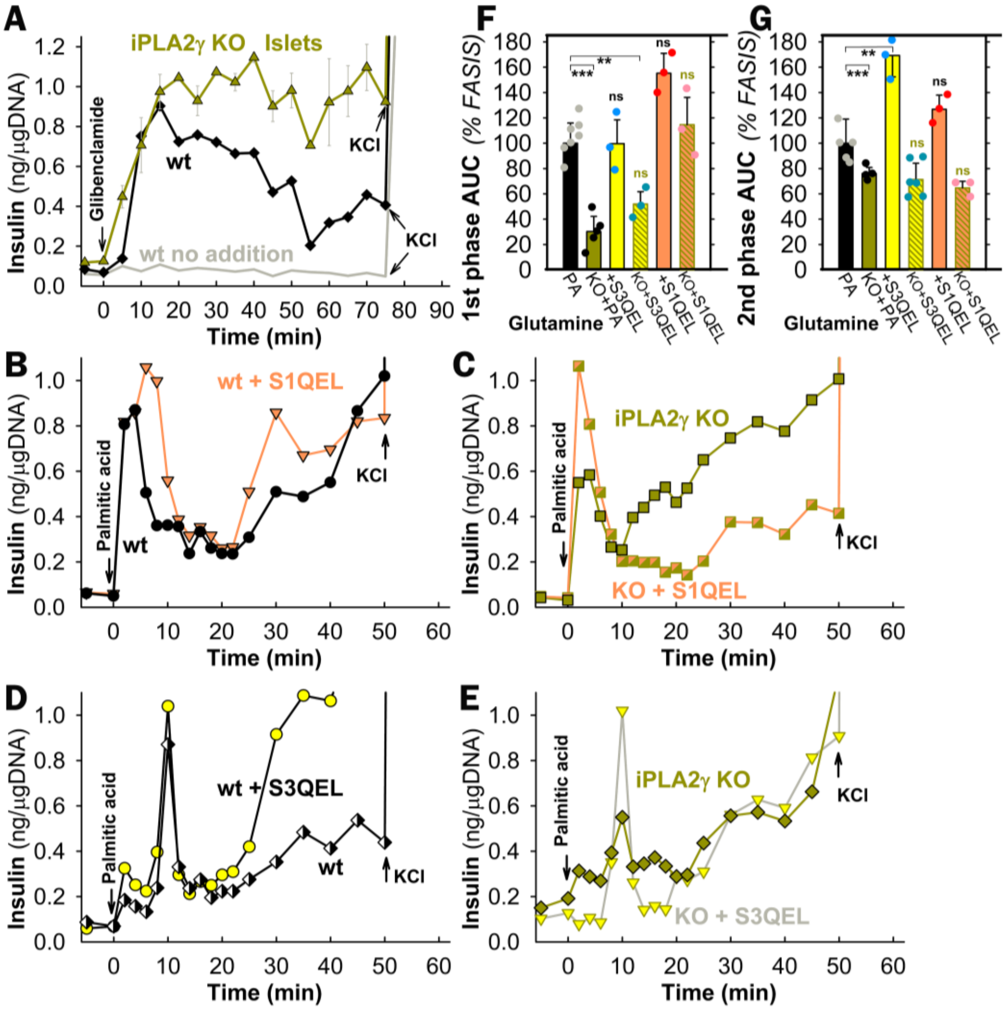

Figure S3. Insulin secretion stimulated with glibenclamide and no effects of suppressors of electron leak on fatty acid-stimulated insulin secretion (FASIS) in isolated pancreatic islets – PIs of backcrossed wt mice (*black, or semi-filled symbols*) and iPLA2γKO mice (*green*) were perifused with KRH medium containing 5.5 mM glucose, while insulin was sampled after additions of (**A**) 50 nM **glibenclamide**; or (**B**–**E**) 2.55 nM free palmitic acid (PA, 50 µM in 0.2% BSA, *i.e.* 30 µM) without: (**B**,**C**) 10 µM **S1QEL** (*orange traces and symbols*); or (**D**,**E**) 10 µM **S3QEL** (black *traces and yellow symbols*). Panels (**F**,**G**) summarize AUCs calculated for the FASIS 1st phase (0–16 min) and 2nd phase (18–50 min), while the background was subtracted as a minimum value. ANOVA of groups („ns“, non-significant *vs.* wt, *black*; *vs.* KO *green*): ** $p < 0.05$; *** $p < 0.001$.

Also, we tested the effects on FASIS of suppressors of 1Q site and 3Q site electron leaks (S1QEL and S3QEL, respectively) (Fig. S3B–G), *i.e.* specific suppressors of superoxide formation within the Complex I site I_Q and Complex III site III_{Qo}, respectively [58,59,62]. Typical PI perifusion records are illustrated (Fig.S3B–E), as well as AUCs (Fig. S3F,G), exhibiting no inhibition, with the exception of a statistically insignificant S1QEL-mediated inhibition of the 2nd phase in iPLA2γKO-PIs.

Part IV Respiration of pancreatic islets from wt *vs.* iPLA2γ KO mice

Respiration rates evaluated by fluxomics: PI respiration was analyzed with an Agilent Seahorse XF 24 analyzer. The data shown are full records for their detailed parts of Fig.3A–C (averaged from *N*=5). The precise quantification of islet respiration is difficult due to the islet heterogeneity, causing different number of cells in each run. But, when the ratios between respiratory rates for a single sample are calculated, possible irregularity in cell number within each islet sample is compensated in this way.

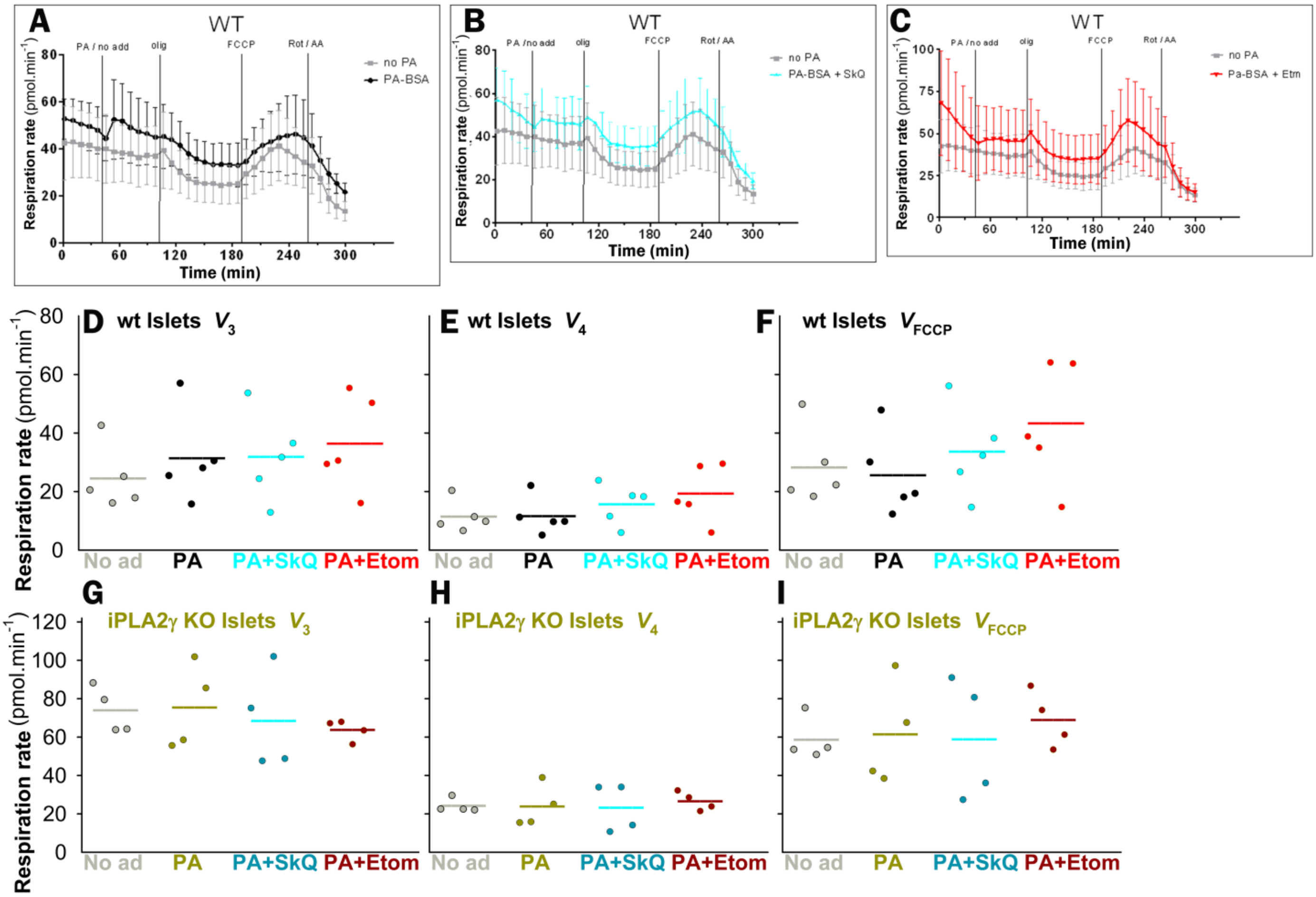

Figure S4 Respiration rates corrected for nonmitochondrial respiration – compared wt *vs.* iPLA2γKO pancreatic islets. **A–C**) Representative records of Agilent Seahorse XF 24 analyzer for pentaplicates (*N*=5) of a typical single islet isolation. **D–I**) Derived respiratory rates, corrected for non-mitochondrial respiration. Panels **A–C**) compare respiration rates in the absence of palmitic acid to the rates **A**) in the presence of 50 μM palmitic acid with 30 μM BSA; **B**) with 10 nM SkQ1 in addition to 50 μM PA (+30 μM BSA) and **C**) with 2.5 μM etomoxir in addition to 50 μM PA (+30 μM BSA). Panels **D–I**) sort the derived respiration rates according to the state, either phosphorylating respiration rates (V_3), non-phosphorylating respiration rates, set with oligomycin (V_4) and roughly derived maximum rates in the presence of carbonyl cyanide-p-trifluoromethoxyphenylhydrazone (FCCP; V_{FCCP}).

Therefore, we present here a typical example of records (Fig. S4A–C) and derived values of pentaplicates of respiratory rates for a single isolation of wt islets (Fig. S4D–F) and iPLA2γ KO islets (Fig. S4G–I). They were selected from altogether *N*=5 isolations of islets and up to *n*=15 single rates evaluations for each condition, which were used to calculate phosphorylating to non-phosphorylating respiration ratios of Fig. 1N. Oligomycin was used to set the non-phosphorylating respiration. The minimum OXPHOS values at 5.5 mM glucose in the absence or presence of etomoxir in iPLA2γKO PIs (Fig. 1N) then reflect the absence of

endogenous FAs cleaved from mitochondria, as expected in the absence of phospholipase. Probably in wt PIs these endogenous FAs cleaved by phospholipase with non-zero activity (but not yet activated to maximum) contribute to the observed higher OXPHOS intensity.

Part V Carnitine palmitoyl transferase 1 silencing

Verification: The Silencer Select siRNA for carnitine palmitoyl transferase 1 (ThermoFisher, ID: s130674 Cpt1a) was verified by PCR for its efficiency 48 hours after transfection. The relative expression ratio (decrease) was calculated only from the real-time PCR efficiencies and the crossing point deviation of an unknown "silenced" sample vs. control parental INS-1E cells and cells transfected with siRNA bearing the scrambled sequence, using formula:

$$\text{Decrease} = (E_{target})^{\Delta \mathbf{CP} target\ (control - sample)} / (E_{ref})^{\Delta \mathbf{CP} ref\ (control - sample)} \quad \{eq.2\},$$

from the reference [73], Pfaffl MW. Nucleic Acids Res. 2001;29(9):e45. E_{target} is the PCR efficiency of target gene transcript, while E_{ref} is the PCR efficiency of the used reference gene. **ΔCP**target is a crossing point deviation between control and sample for the target gene transcript, while **ΔCP**ref stands analogically for reference gene. We performed PCR for a CPT1 amplicon with a forward primer CTGTCAACCTCGGACCCAAA and a reverse primer CAGCATCTCCATGGCGTAGT. From the data obtained by PCR and using the eq.{2}, we have obtained the relative decrease of CPT1 amplicon down to 20% for treatment of INS-1E with s130674 Cpt1a (Fig. S5A). The control housekeeping gen was rat YWHAZ, amplified with a forward primer AGCCCGTAGGTCATCTTGGA and a reverse primer with sequence of TGCGAAGCATTGGGGATCAA. Realtime qPCR was performed on a Biorad CFX Connect, using protocol with 50 cycles, temperatures 95 °C, 59 °C, 72 °C, timing 7 s, 10 s, 15 s, respectively. RNA isolation was done using the RNA Qiagen RNeasy mini kit.

.

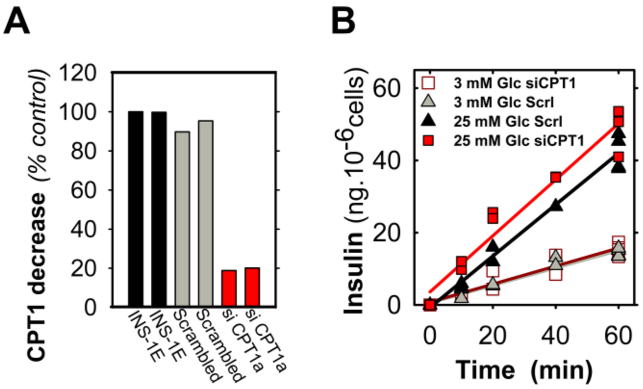

Figure S5. Verification of CPT1 silencing and insensitivity of GSIS to CPT1 deficiency – A) The relative content of CPT1 amplicon, calculated using eq. {2} for parental INS-1E cells (*black*), cells transfected with scrambled siRNA (*gray*) and cells transfected with s130674 Cpt1a. **B) GSIS:** 3 mM (*gray*, *dark red*) or 25 mM glucose (*black*, *red*) was added to INS-1E cells after twice preincubation in KRH with no glucose and accumulated insulin was assayed. The calculated differential rate of insulin secretion (slope of samples with 3 mM glucose subtracted) after CPT1 silencing accounted for 112% of the rate in controls, transfected with scrambled siRNA.

Insensitivity of GSIS to CPT1 silencing: Next, we validated the results of FASIS inhibition after CPT1 silencing by checking any effects of this silencing to GSIS. As Fig. S5B demonstrates, there was no effect to GSIS with added 25 mM glucose after twice washing in KRH without glucose for 15 min.

Part VI Monitoring of cytosolic redox signal upon GSIS *vs.* FASIS and induction of insulin secretion with pro-oxidants

Comparison of H_2O_2 release for different fatty acids and doses: The precise quantification of H_2O_2 release to the PI exterior was performed using the two main modes. In the first mode, we excluded any contact and possible interaction of Amplex UltraRed and horseradish peroxidase (HRP) with the islet surface to prevent possible artifacts. Thus, only after perifusate collection, were Amplex UltraRed and HRP added. The other advantage of this mode was perfect time matching and identity with the perifusion experiments surveying insulin secretion. The comparison of selected FAs at total FA concentrations theoretically yielding ~2.5 nM free FAs (according to Table S2) are shown in Fig. 5E, including empirically adjusted concentrations for lauric, decanoic and hexanoic acids.

The second method used a closed cuvette space, when allowing the contact of Amplex UltraRed and HRP with PIs, continually recording the accumulated Amplex UltraRed fluorescence (Fig. 5F–J). For this method, differential saturated rates of H_2O_2 release to PI exterior are compared in Fig. S6 for given FAs and their doses.

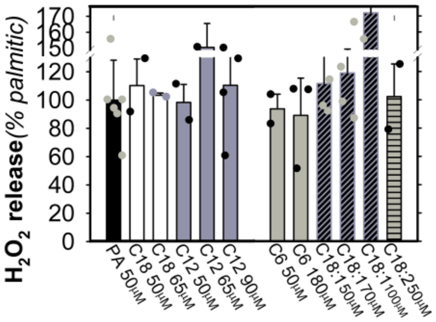

Figure S6 External H_2O_2 release from pancreatic islets monitored with Amplex UltraRed & HRP in a fluorometer cuvette – compared are rates of H_2O_2 release with subtracted rates with no FA added for selected FAs and their indicated total concentrations. The rates were taken when saturated during the time interval of 6 to 10 min and were normalized to those measured with 50 μM palmitic acid (PA)

Validation of Amplex UltraRed method for quantification of H_2O_2 release: The precise quantification of H_2O_2 release to the extracellular space is difficult in a heterogeneous and compact cell cluster like the pancreatic islet. A perifusion method might ensure that a majority of the released H_2O_2 could remain in the perifusate and, if reacted with Amplex UltraRed and HRP in the already collected samples, this neglects any interfering effects of e.g. islets, membranes, and column material. Each 2 min collection of perifusate fluorescence makes it possible to estimate purely the rate of H_2O_2 release to the extracellular space for the

preceding 2 min (5 min after 25 min). Differences between samples with and without PA allowed us to determine differential (Δ) rates, corresponding to a net redox signal.

Also, we have previously demonstrated that NADPH oxidase-4 (NOX4) is the main redox signal source upon GSIS [36]. Now, when introducing the method of H_2O_2 monitoring in the PI cell exterior with Amplex-UltraRed plus HRP, we can validate this method relative to the previously reported H_2O_2 elevation in the cytosol [36]. Indeed, upon the addition of 25 mM glucose to PIs incubated in the cuvette (Fig. 5J), the H_2O_2 release to the islet cell exterior was significantly elevated and was almost entirely dependent on the presence of NOX4 (Fig. 5J). Note that the C57BL/6J progeny was used for the construction of NOX4 KO null mice, unlike a current progeny based on C57BL/6N mice and employed for the construction of iPLA2γKO mice.

Hence, separate "wt" controls backcrossed for 10 generations were used [36] for experiments of Fig. 5J. The saturation most probably occurred due to a limited cuvette volume. With this other control PIs (Fig. 5J), isolated from backcrossed mice based on C57BL/6J progeny the H_2O_2 release saturated to 27 ±2 pmol·min^{-1}·10^{-6} PI cells (N=3), while in NOX4 KO PIs to 12±0.8 pmol·min^{-1}·10^{-6} PI cells (N=3), and the rate prior to glucose in NOX4 KO PIs was 9±0.7 pmol·min^{-1}·10^{-6} PI cells, hence the glucose-dependent H_2O_2 release was negligible.

Indirect validation of Amplex UltraRed method for quantification of H_2O_2 release: We also used data of Fig. 5L together with the quantified respiration of INS-1E cells [37,63] to calculate the % yield of H_2O_2 *vs*. consumed oxygen, which was 0.47% ±0.2% of oxygen without PA and increased to 3% ±0.4% with PA (Fig. S7A). This represents an expected reasonable value, showing that at least the order of magnitude is valid for estimated H_2O_2 release rates. For islets we obtained the same order of magnitude when we took data such as Fig.S4 and Fig.5. Being unable to assay respiration and H_2O_2 simultaneously, we omit such data.

Monitoring of the cytosolic redox signal: Upon FASIS, INS-1E cells also exhibited cytosolic H_2O_2 release, monitored with DCF (Figure S7B,C), or with HyPer-7 (Fig. S7E). Since the DCF probe is a relatively nonspecific ROS indicator, sensing ROS downstream of H_2O_2, we omitted its calibration. Nevertheless, 10 nM SkQ1 inhibited its increasing fluorescence after PA addition (with 15 µM BSA), indicating the absence of the redox signal (Fig. S7B,C). The redox signal was also absent with only 1 µM Agonist II without FA (Fig. S7B–D). Also, INS-1E cells transfected with the cytosolic HyPer-7 fluorescence probe yielded minor, but statistically significant, increases in fluorescence intensity ratios at 488 nm *vs*. 405 nm upon a standard cell FASIS test (15 µM palmitic acid, with 15 µM BSA) (Fig. S7E).

Rescue of cytosolic redox signal: Previously, we also demonstrated that the lack of NOX4, disabling GSIS, can be rescued with the externally added H_2O_2 to PIs of NOX4 KO mice (cf. Figure 4M,N in [36]). Also, insulin release in INS-1E cells induced by *tert*-butylhydroperoxide (tBHP) reached ~70% of GSIS (cf. Fig. 7B in [37]). In iPLA2γ-silenced INS-1E cells, the tBHP-induced insulin release was even higher due to the absence of antioxidant uncoupling otherwise provided by a synergy of UCP2 plus iPLA2γ [37]. We conducted similar experiments in wt and iPLA2γKO-PIs using H_2O_2 (Fig. 5N–P). We also present here the induced response of PIs by tBHP, manifested as a relatively low 1st phase of insulin release in wt-PIs but not iPLA2γKO-PIs; followed by a delayed "rescue" at 30 min in both wt as well as in iPLA2γKO-PIs (Fig. S7F). A negligible inhibition by GW1100 was observed for such a "rescue" by H_2O_2 (Fig. S7G), while AUCs for both phases taken together are listed in Fig. S7H (including more data as well as the data of Fig. 5N–P; *N*=3–6).

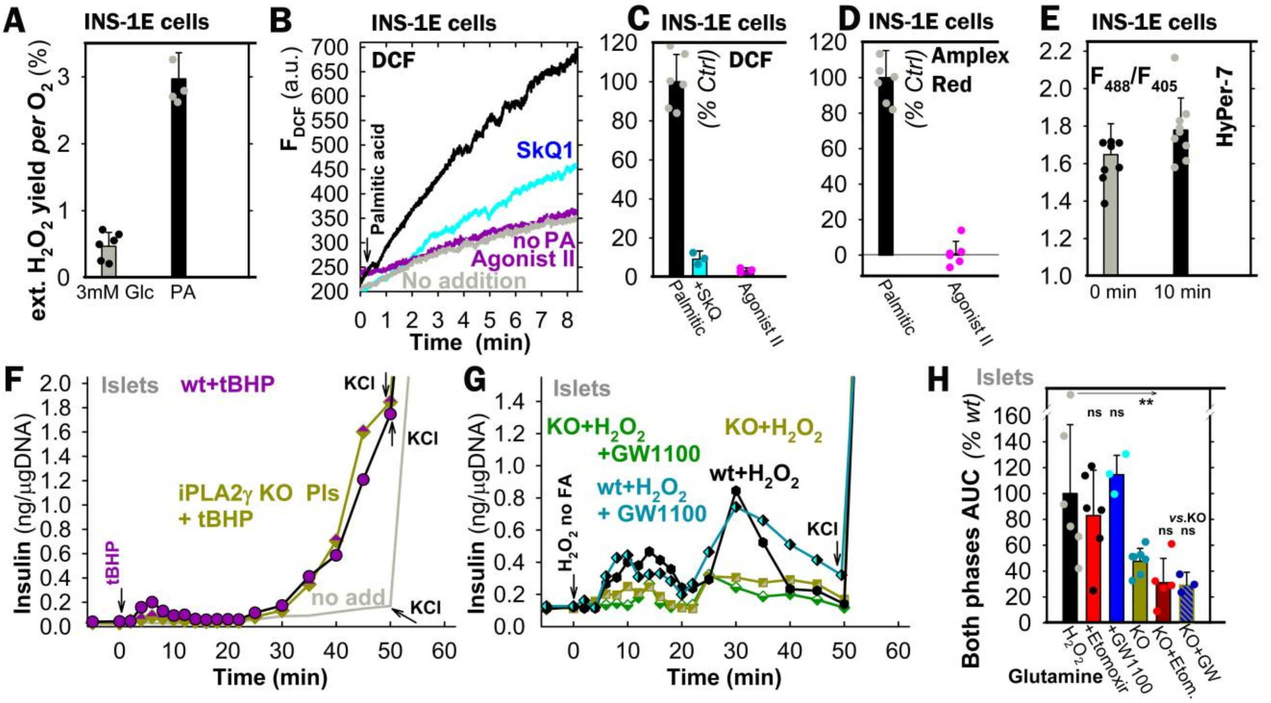

Figure S7 Monitoring of cytosolic redox signal upon FASIS, and induction of insulin secretion with pro-oxidants – wt PIs *vs*. iPLA2γKO PIs as indicated. (**A**) **Extracellular H_2O_2 release rates as % of oxygen consumed by respiration** – data from Fig. 5L were used together with the quantified respiration of INS-1E cells[37,63] to calculate % yield of H_2O_2 *vs*. consumed oxygen (values of 24 ±6 $O_2 \cdot s^{-1} \cdot 10^{-6}$cells, *i.e*. 1416 ±378 $O_2 \cdot min^{-1} \cdot 10^{-6}$cells were used for no FA added; 1416 for PA). **B**,**C**) DCF monitoring of cytosolic H_2O_2 release upon FASIS (15 µM palmitic acid, i.e. 1.4 nM free with 15 µM BSA) in suspension of INS-1E cells. Typical records (**B**) and quantifications of inhibition (**C**; when "no addition rates" were subtracted). Where indicated, 10 nM SKQ1 together with PA (*cyan*) or 1 µM Agonist II without PA were used. Note that the sole 1 µM Agonist II had no effect even when using exterior H_2O_2 release monitoring with Amplex UltraRed (**D**). (**E**) HyPer-7 fluorescence intensity ratios at 488 nm *vs*. 405 nm in standard cell FASIS test (15 µM palmitic acid, i.e. 1.4 nM free with 15 µM BSA) prior to (0 min) and 10 min after palmitic acid addition. Data collection from 9 cell passages (averages for each biological replicate are indicated) containing estimations of 66 cells. **F**–**H**) **Insulin secretion stimulated by *tert*-butylhydroperoxide (tBHP) (F) and H_2O_2 (G,H) at low glucose (5.5 mM)** – wt-PIs (*black*, *violet*) and iPLA2γKO PIs (*green, green/violet semifilled symbols*) were perifused in the presence of 100 µM tBHP (**F**) or 100 µM H_2O_2 (**G**,**H**). Where indicated, 1 µM GW1100 (*black/cyan* or dark *green/white* semi-filled symbols) was present (**G**); or 2.5 µM etomoxir (*red*, *dark red*) was tested (**H**).

Part VII Responses of Ca^{2+} oscillations to glucose in wt *vs*. iPLA2γKO murine PIs

We compared oscillations in cytosolic Ca^{2+} responding to palmitic acid (Fig. 7) to those at increasing glucose between 3 mM and 20 mM, monitored in cells with an expressed slow variant of the GCaMP6 fluorescence probe by confocal microscopy (Fig. S8). Fluorescence emission was collected from ROI within each single responding cell. Observed cytosolic Ca^{2+}-oscillations $[Ca^{2+}]_c(t)$ at a given increasing glucose differed in peak intensity histograms. At 3 mM glucose, over 60% of peaks were of the smallest intensity, falling into the lowest decile (*i.e*. 10-percentile) of the whole intensity range, with other peaks falling in the second and third smallest bin. At 5 mM glucose, the $[Ca^{2+}]_c(t)$ spikes with ~ 20% of maximum intensity were most frequent and at 9 mM glucose those with ~ 50% of maximum intensity were most frequent, while in both cases even higher intensities were abundant.

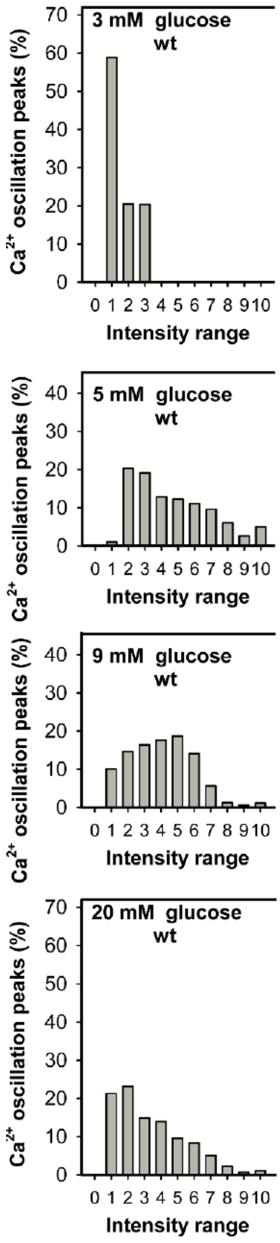

Figure S8A Histograms of intensities of Ca^{2+}-oscillations, binned in deciles for INS-1E cells, induced with increasing glucose concentrations, indicated in each panel. Around 1000 Ca^{2+}-oscillation peaks were taken.

Figure S8B Theoretical histograms of intensities of Ca^{2+}-oscillations for nimodipine and exp. results of catalase overexpression, binned in deciles for INS-1E cells. Here the first decile was divided into noise (below 5% of the maximum signal) and the remaining first decile. Around 20 min of single-cell records were analyzed with nimodipine plus PA, and 10 min with PA and catalase overexpression.

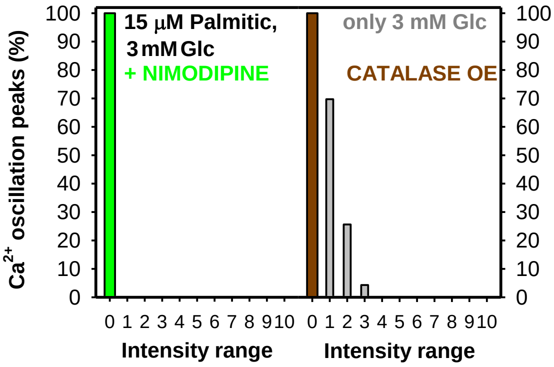

Part VIII GSIS and absence of insulin resistance in iPLA2γKO mice

GSIS was not significantly different in wt and iPLA2γKO mice (Fig. S9A–D), while the knock-outs did not exhibit any peripheral insulin resistance (Fig. S9E,F). GSIS was tested in mice either after i.p. glucose administration, as described previously [36] (Fig. S9A,B), or after oral glucose administration (Fig. S9C,D). Statistically, with a low significance, the GSIS 2nd phase in iPLA2γKO mice appeared to be somewhat higher (Fig. S9C), which may reflect a mild impaired glucose tolerance.

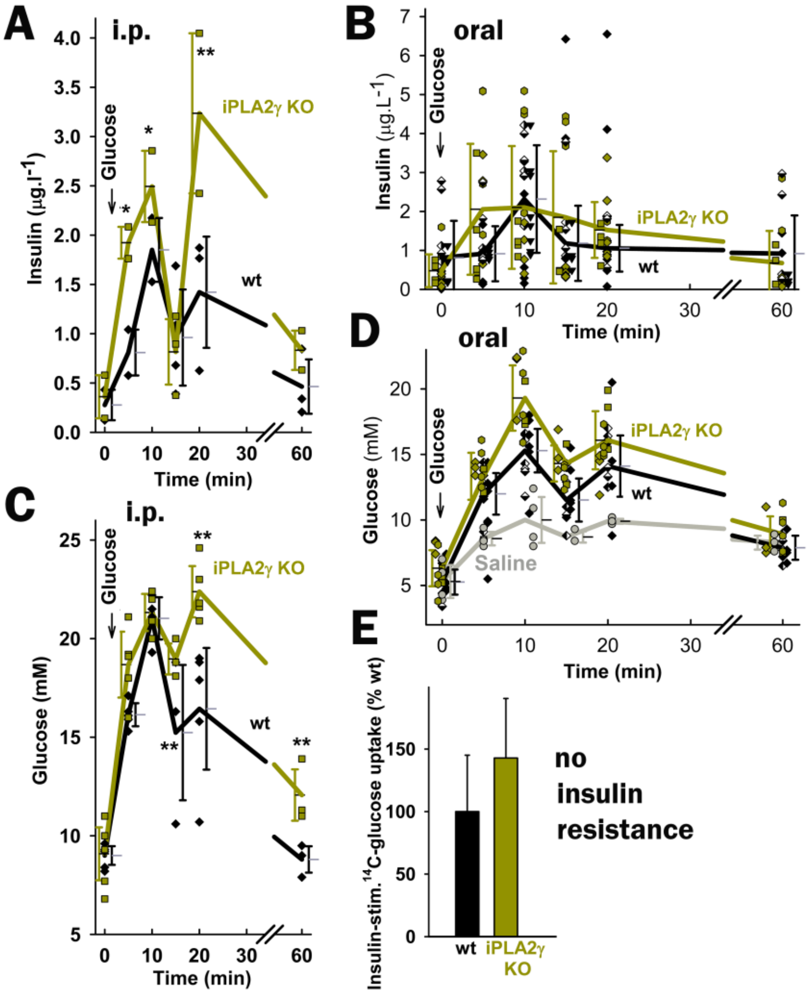

Figure S9. GSIS in wt *vs.* KO mice – intraperitoneal (i.p.) administration of glucose (1 mg/g body weight; ~111 μmol glucose *per* mice) (**A**,**C**) or oral administration of glucose (1 mg/g body weight) (**B**,**D**) was performed with wt (*black*) and iPLA2γ/PNPLA8 KO mice (*green*). At 2–3 time points (*i.e.* estimates of insulin and glycemia, organized so to cover 60 min), blood was sampled from the eye plexus blood vessel for each mouse and resulting insulin time courses (**A**,**B**) and glycemia time courses (**C**,**D**) were constructed. Average values are connected by thick lines, while all measured time points of several experiments are shown. The time dependencies were constructed from numerous groups of mice of different littermates, both males and females (9 and 32 mice in each group for **A**,**C** and **B**,**D**, respectively) covering all the tested time points, while the different littermates are indicated with different symbols. This setup is the only way to perform fast sampling of insulin release using the most sensitive insulin Elisa kit (Mercodia, Uppsala, Sweden). Student's T-test calculated for each time point individually yielded non-significant differences except for pairs marked with: ** $P < 0.05$; * $P < 0.1$. In panel D, all data up to 20 min were statistically significant ($P < 0.05$) relative to saline-only data.

Figure S9E Insulin resistance was assayed as ^{14}C-glucose uptake into lipids of epidydimal adipose tissue (for method, see Ref. [36]), sensitive to insulin (differences between with and without insulin were assessed; *n* = 9).

For i.p. administration, the 1st GSIS phase peaked at around 10 min in wt (backcrossed) mice (Fig. S9A) and was slightly higher ($P < 0.05$) in iPLA$_2$γKO mice (AUC^{1st} 148%–162% of wt AUC^{1st}). The 2nd GSIS phase was even higher in some individual iPLA$_2$γKO mice (AUC^{2nd} 257%–284% of wt AUC^{2nd}).

With glucose administered orally, *i.e*. providing a test that also includes responses to incretins, the data for an ensemble of iPLA$_2$γKO mice yielded an overall earlier initial insulin rise with a wider peak relative to wt mice, in which a sharper peak of the GSIS 1st-phase was observed. The 2nd phases clearly exhibited glycemia (Fig. S9C,D), decaying similarly in wt and iPLA$_2$γKO mice.

These data already suggest that no significant glucose intolerance was developed in iPLA$_2$γKO mice, despite slightly but statistically insignificantly higher values than wt mice (Fig. S9B). When assayed as the insulin-induced ^{14}C-glucose uptake into epidydimal white adipose tissue of iPLA$_2$γKO *vs*. wt mice, no significantly developed insulin resistance was found (Fig. S9E).

Part IX Details of *in vivo* FASIS in wt *vs*. iPLA2γKO mice

First, we will discuss whether three apparent phases of insulin secretion after oral administration of Intralipid to mice are only delayed in iPLA$_2$γKO mice. We have calculated AUCs from the data of Fig. 9A, while taking averages ± SDs and recognizing three phases for wt mice (0–20 min; 20–240 min; and >240 min) and four phases for iPLA$_2$γKO mice (0–15 min; 15–60 min; 60–240 min; and >240 min) (Fig. S10A). This sectioning complies with the assumption that FASIS is only delayed in iPLA$_2$γKO mice, specifically the 1st wt phase is shifted to the 15–60 min of iPLA$_2$γKO mice.

Second, the ratio of molecular weights for murine c-peptide (3121.41) *vs*. insulin (5803.6) is 0.537840306. Calculating this ratio for their estimated values for each individual mouse provides more precise insight into physiological postprandial events. Whenever this ratio exceeds the true stoichiometry of 0.5378, insulin was internalized/degraded faster from the blood relative to c-peptide. This internalization is supposed to be maximum when an insulin secretion initiates again. Hence, the co-incidence of c-peptide to insulin peaks with the insulin peaks supports the new starting insulin secretion at the given moments.

Third, in order to determine whether insulin secretion stimulated upon the initial administration of either palmitic acid (Fig. 8A,C), Intralipid (Fig. 8B,D,F; Fig. 9A–E), or Agonist II (Fig. 8I) may contain a component given by the endogenously elevated glucose exceeding the initial fasting levels in mice, we recalculated experimental data of blood insulin and glycemia to divide the secreted insulin amounts by the glycemia in the given period (units in ng *per* mmol). When the glucose (glycemia) does not contribute to insulin secretion, the resulting time course is like that for insulin alone. This was specifically the case for longer periods from the initial oral lipid administration (Fig. S10A). With Agonist II i.p. administration, data reflect its modest ability to stimulate insulin secretion at low glucose (Fig. S10B,C). With GSIS, normalized peaks of insulin secretion are only transformed into small elevations of insulin/glycemia values (Fig. S10D,E).

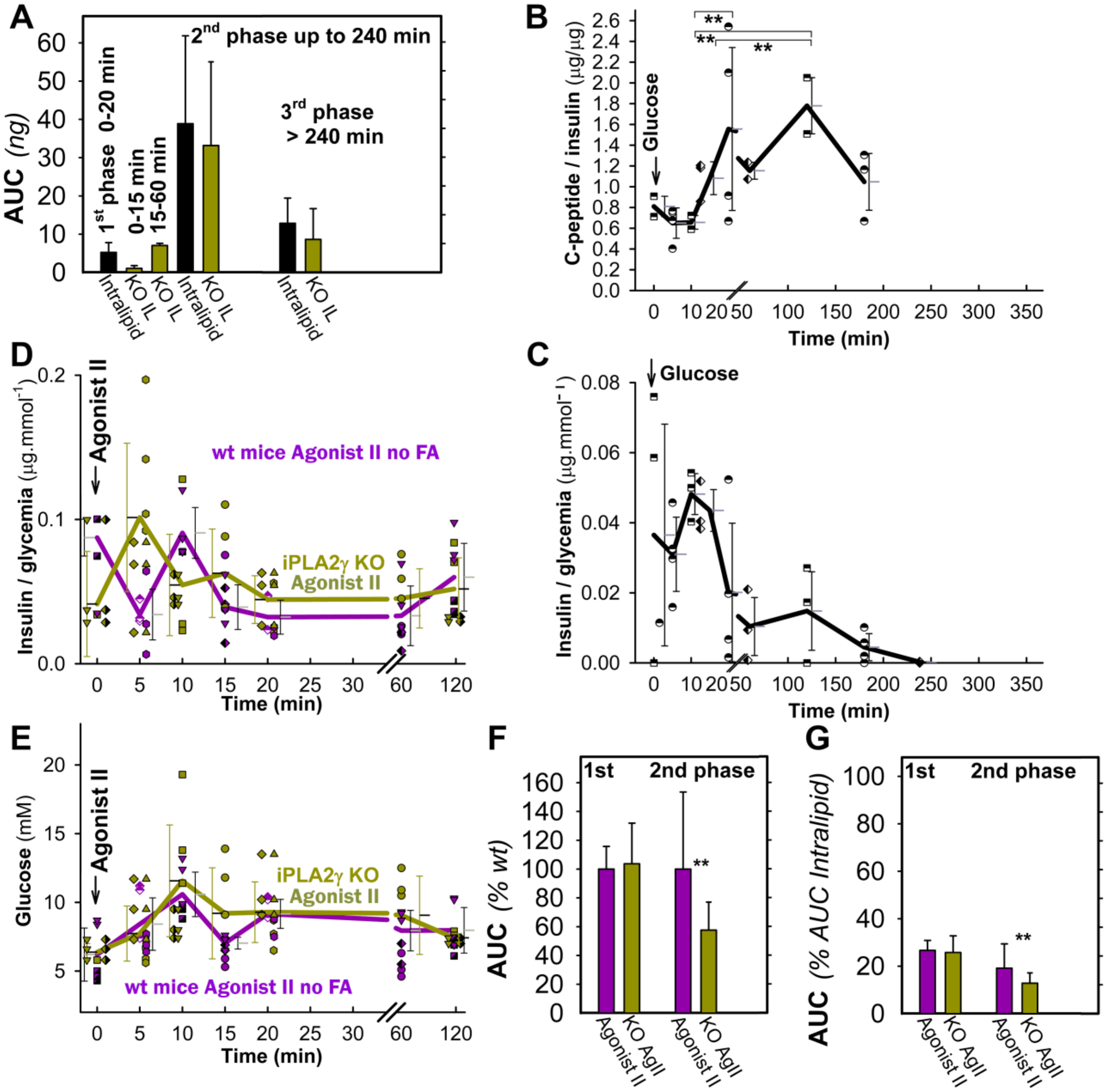

Figure S10. Parameters of insulin secretion stimulated in mice with oral Intralipid and i.p. administered Agonist II – (**A**) AUCs for insulin time courses up to 360 min in three phases after oral administration of Intralipid (data of Fig. 9A); (**B, C**) GSIS (i.p. glucose) for comparison – parameters derived as C-peptide to insulin ratios (**B**) and insulin to glycemia ratios (**C**) from the data of Fig. 9D–F. **D** – **G**) Parameters of insulin secretion in mice after i.p. administration of Agonist II (data of Fig. 8I).

Part X Derived conditions for insulin secretion

We can derive the conditions required for insulin secretion as follows: at least two conditions must be met (Table S3): *i*) there must be a redox signal (elevated cytosolic ROS release) plus cytosolic or *peri*-plasma-membrane ATP (ATP/ADP) elevations; or, alternatively, *ii*) proper phosphorylation of channels (K_{ATP}, TRPM, CaL) must be ensured [39–41,43–45].

	Cytosolic H_2O_2 **elevation**	**ATP** (ATP/ADP) **elevation**	**Phosphorylation of channels** **PIP_2 release** from K_{ATP}
Low glucose	-	-	-
FASIS	++	+	+
FASIS, iPLA2γ KO mice	++	+	-
GPR40 Agonist II	-	-	+
GSIS	+ by NOX4	+	-
GSIS + GLP-1	+ by NOX4	+	+
BCKA IS	++	+	-

Table S3. Two conditions must be met for insulin secretion stimulated with various secretagogues – **Low glucose**: there is only a relatively high mitochondrial superoxide release to the matrix [37,62] (Fig. 4A), which is unable to stimulate insulin secretion without ATP (ATP/ADP) elevation and the receptoric pathways. **FASIS**: both conditions are met. FA β-oxidation provides redox signaling spread from the mitochondrial matrix up to the plasma membrane. The FA-β-oxidation also supplies elevated ATP. The redox signaling also activates phospholipase iPLA2γ/PNPLA8, which cleaves mitochondrial FAs. These mitochondrial FAs predominantly activate the GPR40 metabotropic receptor pathway, enabling the canonical Gαq/11 pathway to act via the Ca^{2+}-dependent PLC-hydrolysis of PIP_2 ("PIP") [24–33], releasing PIP_2 from its binding site on K_{ATP} and abolishing its permanent opening[47]. Hydrolyzed DAG and IP3 provide branching into pathways incorporating PKC and ER-(IP3R), while PKC phosphorylates TRPM4 and TRPM5 channels [45], enabling them to cooperate in triggering of the Ca_V-opening cycles. Biased GPR40 activation can initiate the Gαs-PKA (or EPAC2) pathways, phosphorylating TRPM2 [39–45], K_{ATP}, and Ca_V (or Munc13-1 and ryanodyne receptor [39] on ER). The receptoric pathways are missing in iPLA2γKO mice. **A GPR40 non-metabolizable agonist alone**, such as Agonist II, allows the release of PIP_2 from its binding site on K_{ATP} and hence the triggering of relatively less intensive insulin exocytosis. A high mitochondrial superoxide release to the matrix is not projected to the elevation of cytosolic H_2O_2. **GSIS**: NOX4 produces the redox signal [36], while glucose metabolism by OXPHOS produces ATP. **GSIS + incretins**, such as GLP-1, meet both conditions like FASIS, since GLP-1 and other incretin receptors employ the above-described Gαs-PKA (or EPAC2) pathways. **BCKA IS**: 2-keto-isocaproate (KIC) or 2-ketoisovalerate or 2-ketomethylvalerate, *i.e.* branched-chain ketoacids (BCKAs) produce BCKA-stimulated insulin secretion so that their β-like-oxidation produces a strong redox signal (unaffected by uncoupling protein-mediated attenuation of superoxide formation, synergized by iPLA2γ-mediated FA cleavage [37]) as well as sufficient ATP.
